# Supplementary material for: Synthesis of 3,5-Bis(trifluoromethyl)phenyl-Substituted Pyrazole Derivatives as Potent Growth Inhibitors of Drug-Resistant Bacteria
Source: Molecules. 2021 Aug 22;26(16):5083. doi: 10.3390/molecules26165083 (PMC8398255; doi:10.3390/molecules26165083)
Supplement: Supplementary file 1 [file molecules-26-05083-s001.zip › molecules-1349563-supplementary.pdf]

Synthesis of 3,5-Bis(trifluoromethyl)phenyl-Substituted Pyrazole Derivatives as Potent Growth Inhibitors of Drug-Resistant Bacteria

Ibrahim S. Alkhaibari[1], Hansa Raj KC[1], Subrata Roy[1], Mohd. K. Abu-gazleh[1], David F. Gilmore[2], Mohammad A. Alam[1]*

[1] Department of Chemistry and Physics, The College of Sciences and Mathematics, Arkansas State University, Jonesboro, AR, United States

[2] Department of Biological Sciences, The College of Sciences and Mathematics, Arkansas State University, Jonesboro, AR, United States.

- Correspondence: malam@astate.edu

Characterization data (I-B)

4-[3-[3,5-Bis(trifluoromethyl) phenyl]-4-formyl-pyrazol-1-yl] benzoic acid (III).

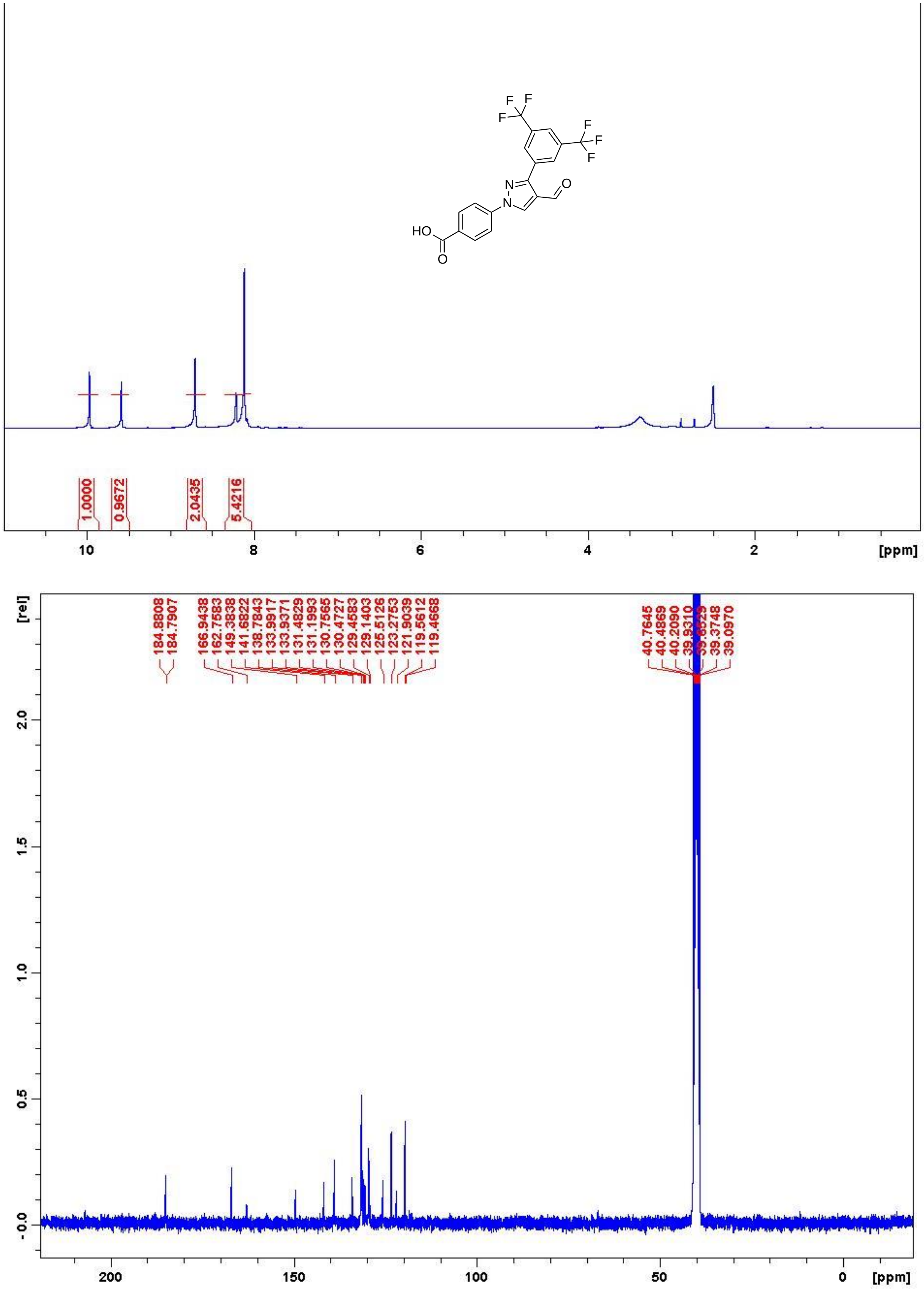

Figure S1. ^{1}H NMR and ^{13}C NMR spectra of compound **III**

4-[4-(Anilinomethyl)-3-[3,5-bis(trifluoromethyl) phenyl] pyrazol-1-yl] benzoic acid (1).

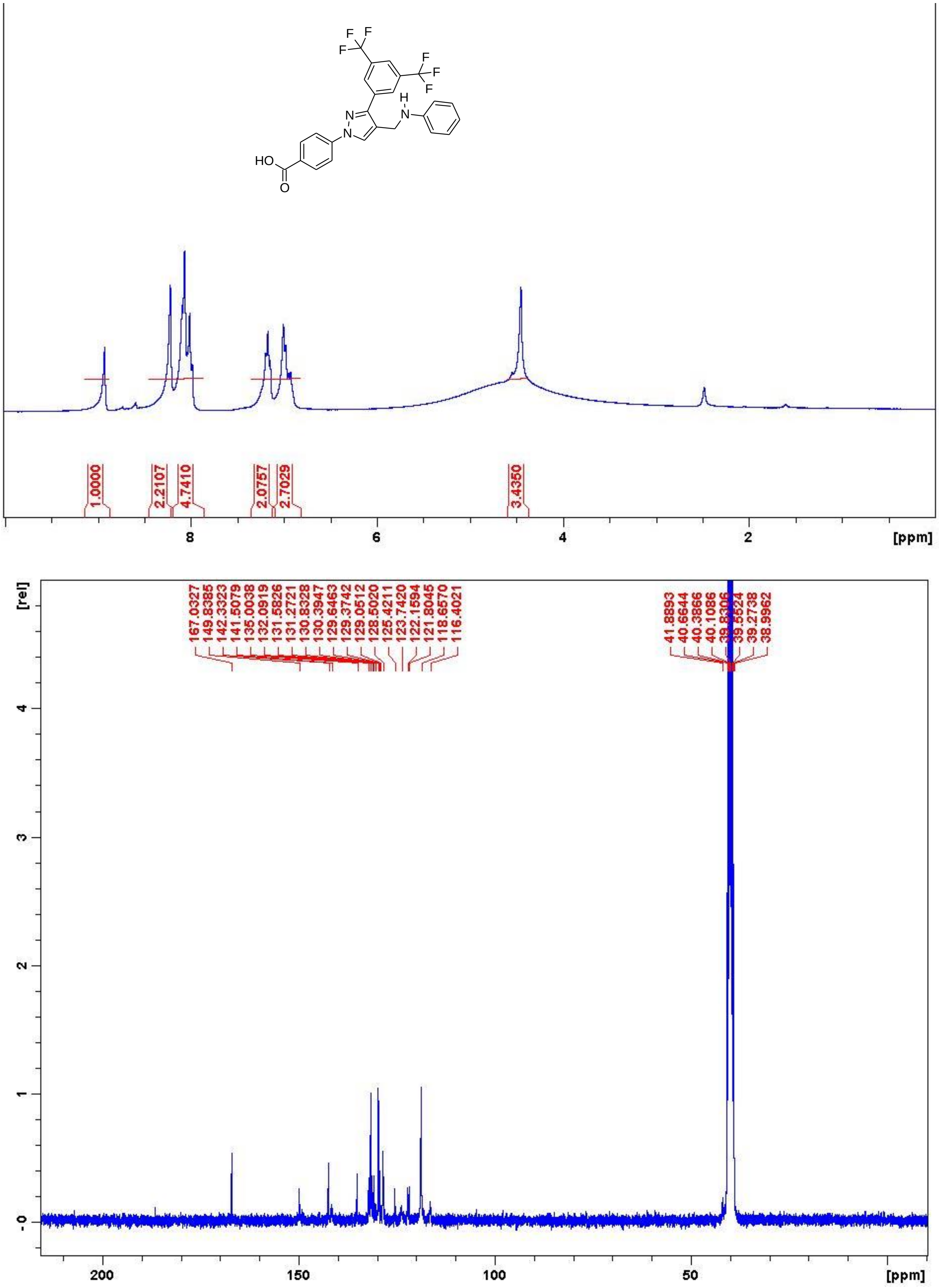

Figure S2. ^{1}H NMR and ^{13}C NMR spectra of compound **1**

4-[3-[3,5-Bis(trifluoromethyl) phenyl]-4-[(4-isopropylanilino) methyl] pyrazol-1-yl] benzoic acid (2).

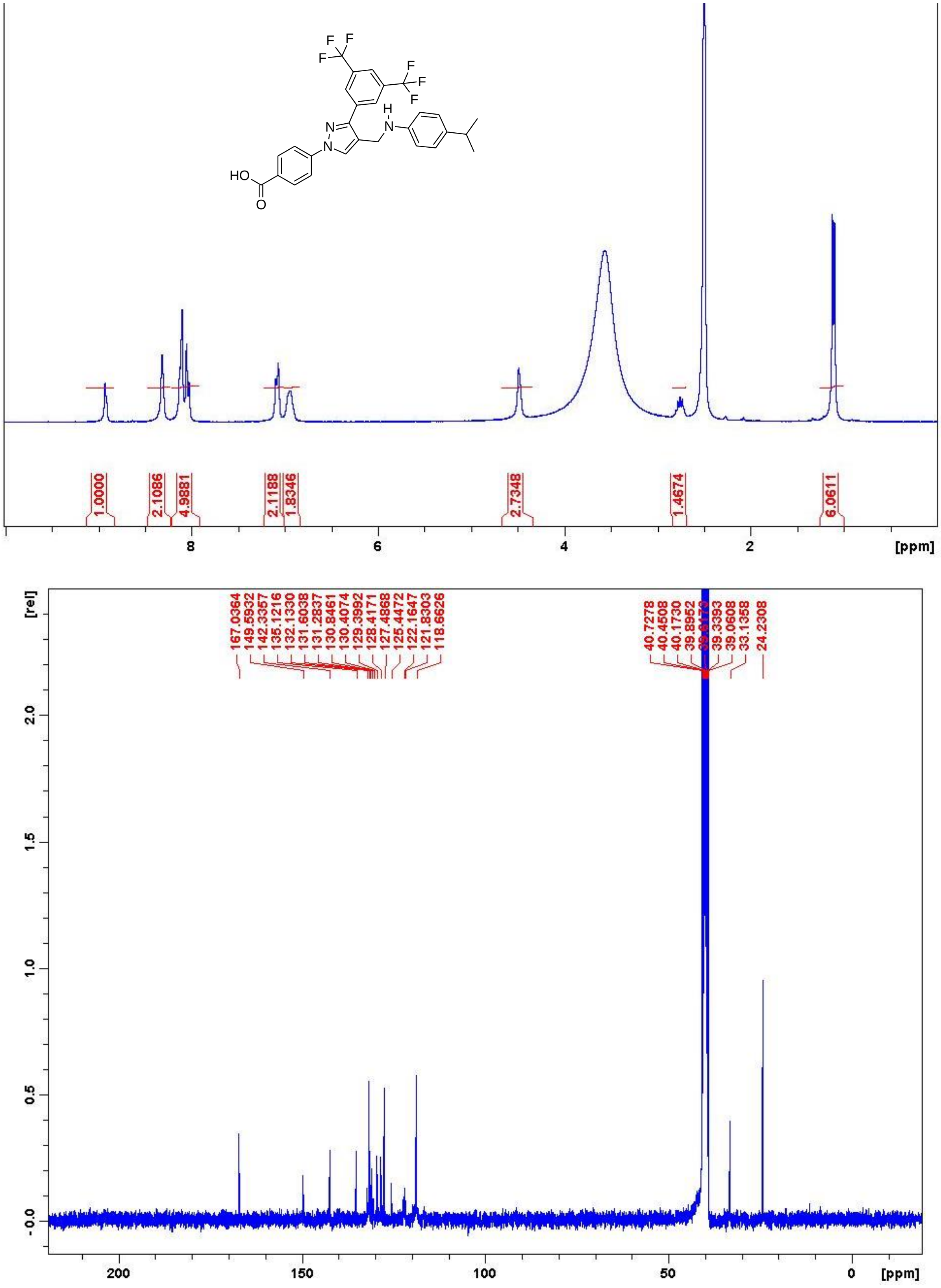

Figure S3. ^{1}H NMR and ^{13}C NMR spectra of compound **2**

4-[3-[3,5-Bis(trifluoromethyl) phenyl]-4-[(4-tert-butylanilino) methyl] pyrazol-1-yl] benzoic acid (3).

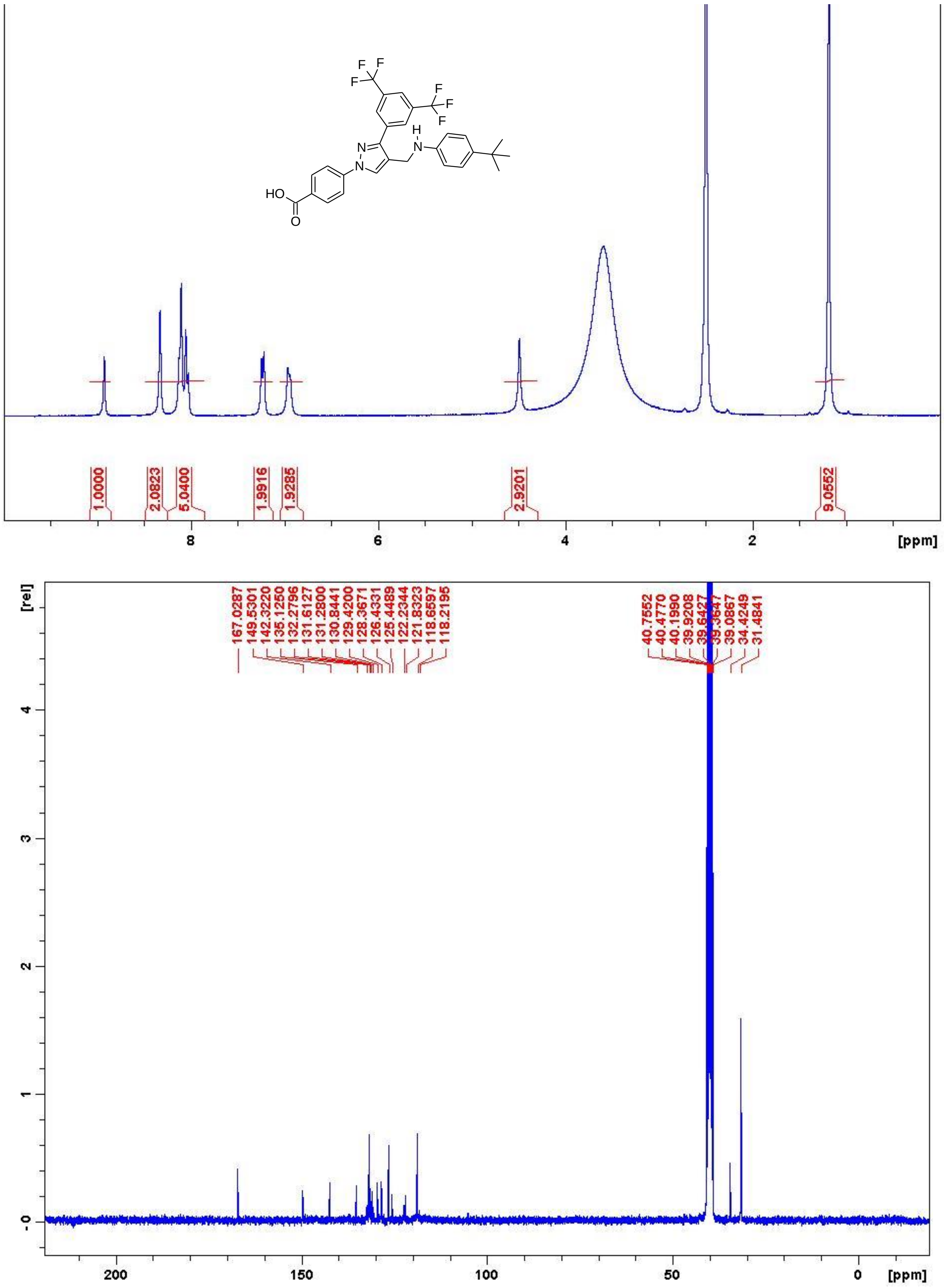

Figure S4. ^{1}H NMR and ^{13}C NMR spectra of compound **3**

4-[3-[3,5-Bis(trifluoromethyl) phenyl]-4-[(4-methoxyanilino) methyl] pyrazol-1-yl] benzoic acid (4).

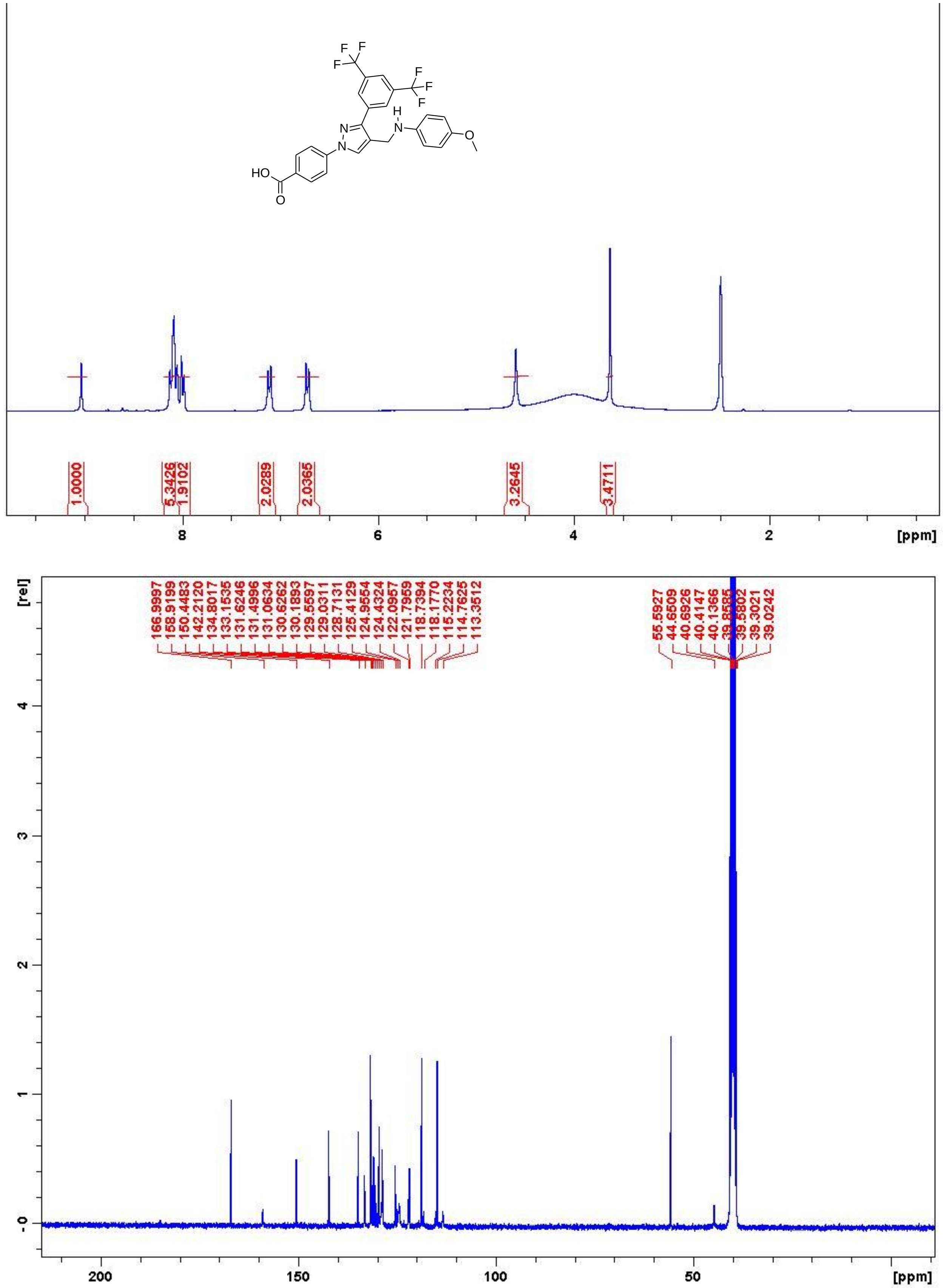

Figure S5. ^{1}H NMR and ^{13}C NMR spectra of compound **4**

4-[3-[3,5-Bis(trifluoromethyl) phenyl]-4-[(4-phenoxyanilino) methyl] pyrazol-1-yl] benzoic acid (5).

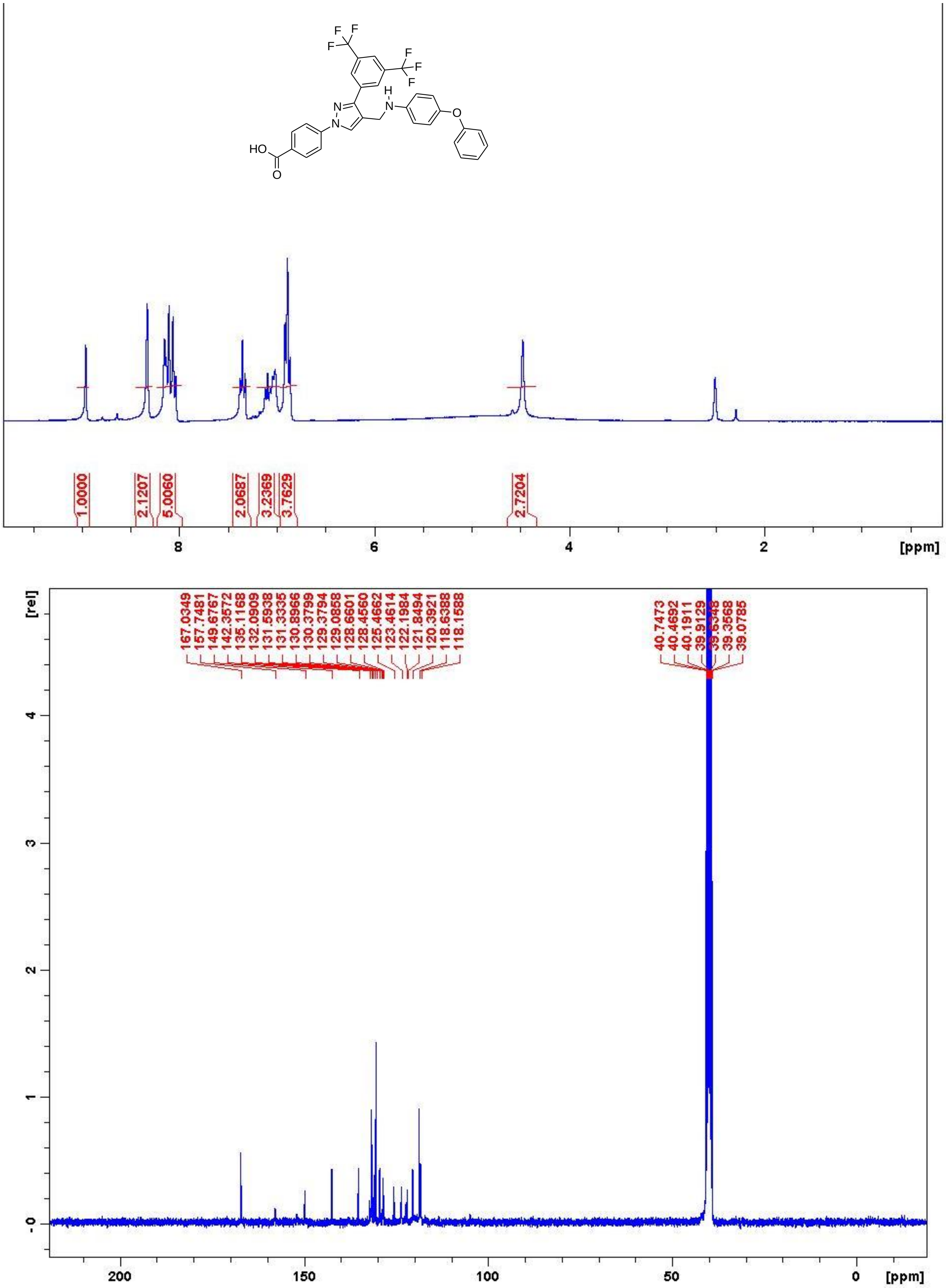

Figure S6. ^{1}H NMR and ^{13}C NMR spectra of compound **5**

4-[3-[3,5-Bis(trifluoromethyl) phenyl]-4-[(4-methylsulfanylanilino) methyl] pyrazol-1-yl] benzoic acid (6).

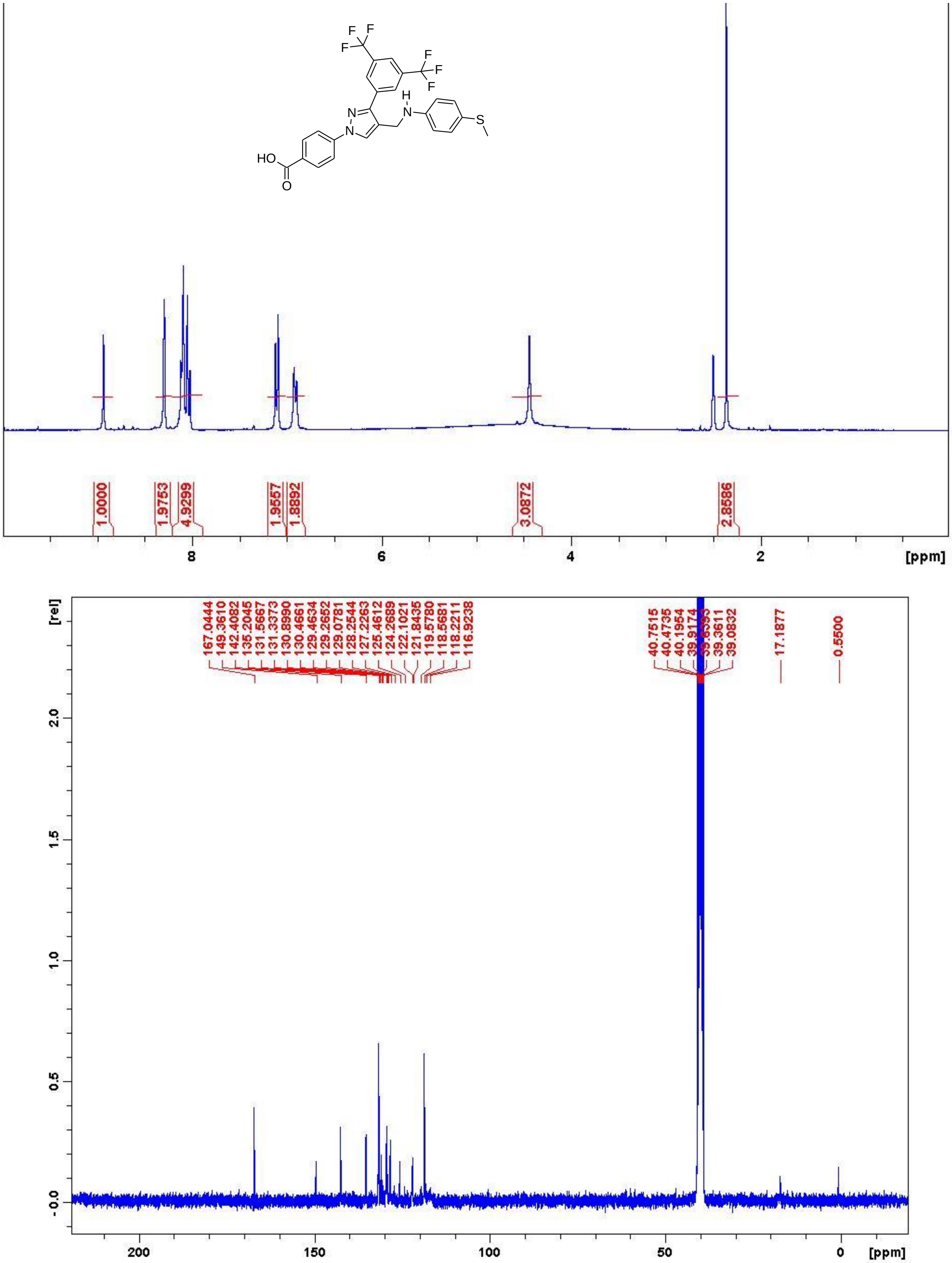

Figure S7. ^{1}H NMR and ^{13}C NMR spectra of compound **6**

4-[3-[3,5-Bis(trifluoromethyl) phenyl]-4-[(3-fluoroanilino) methyl] pyrazol-1-yl] benzoic acid (7).

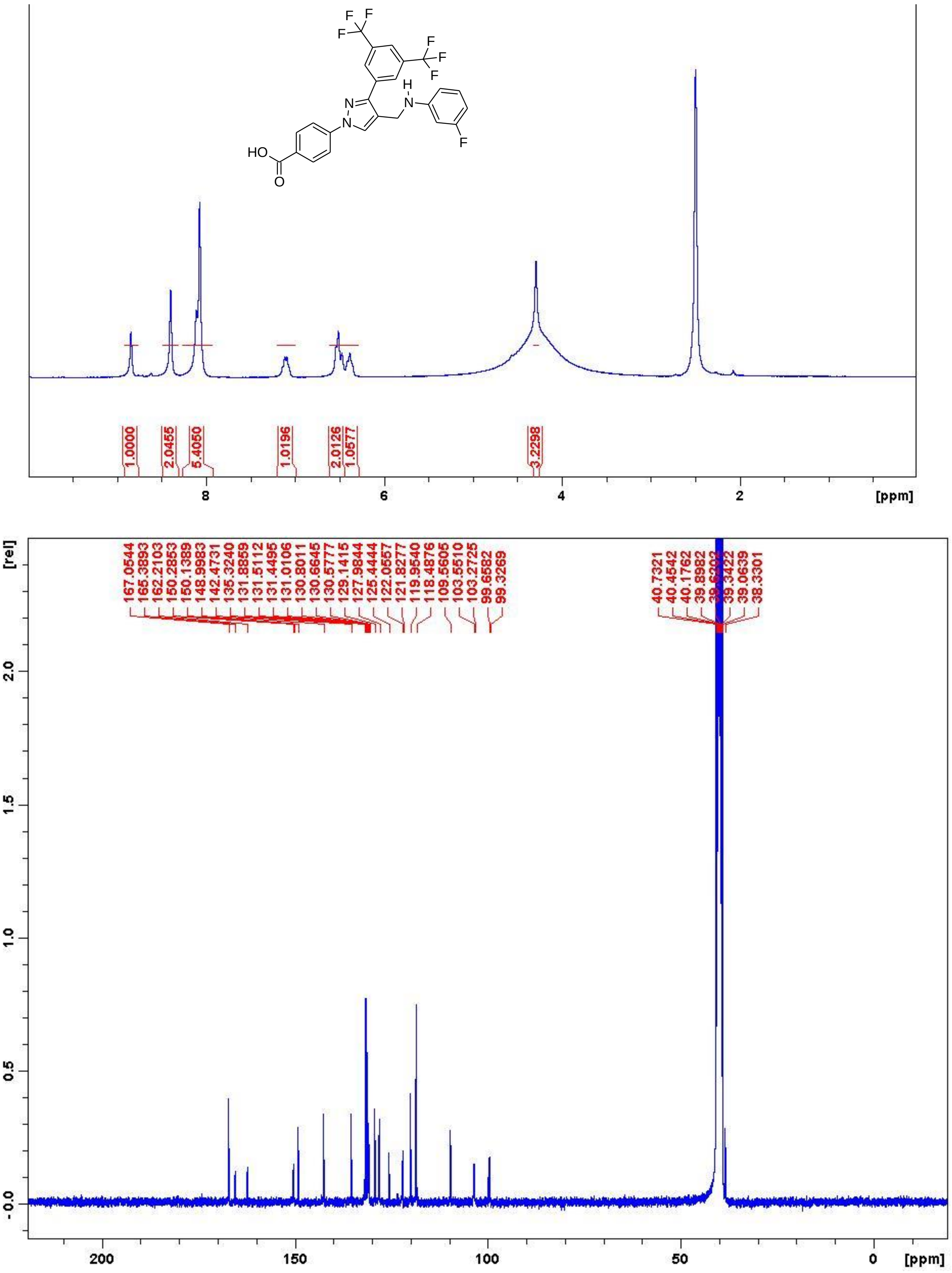

Figure S8. ^{1}H NMR and ^{13}C NMR spectra of compound **7**

4-[3-[3,5-Bis(trifluoromethyl) phenyl]-4-[(4-fluoroanilino) methyl] pyrazol-1-yl] benzoic acid (8).

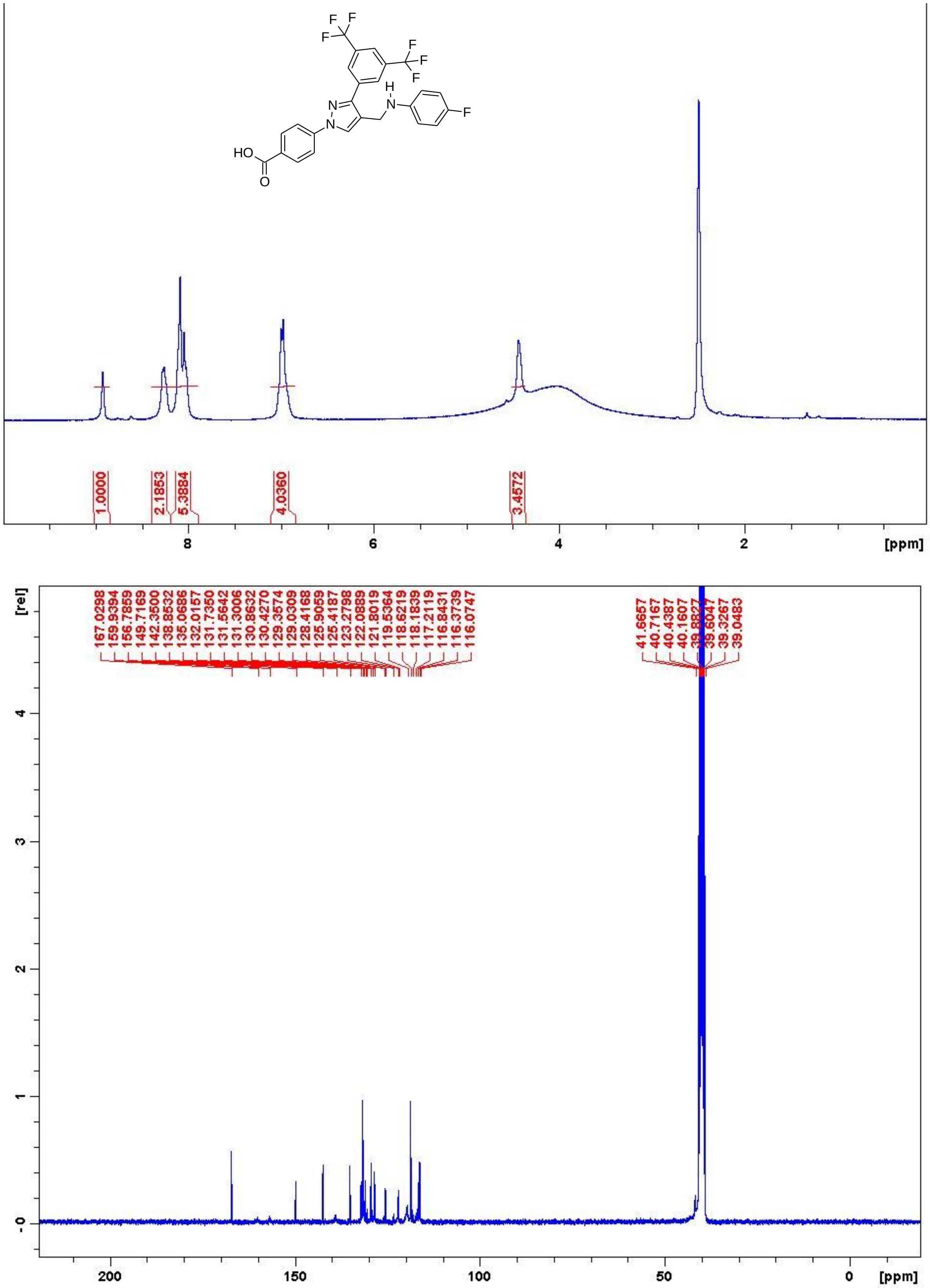

Figure S9. ^{1}H NMR and ^{13}C NMR spectra of compound **8**

4-[3-[3,5-Bis(trifluoromethyl) phenyl]-4-[(4-chloroanilino) methyl] pyrazol-1-yl] benzoic acid (9).

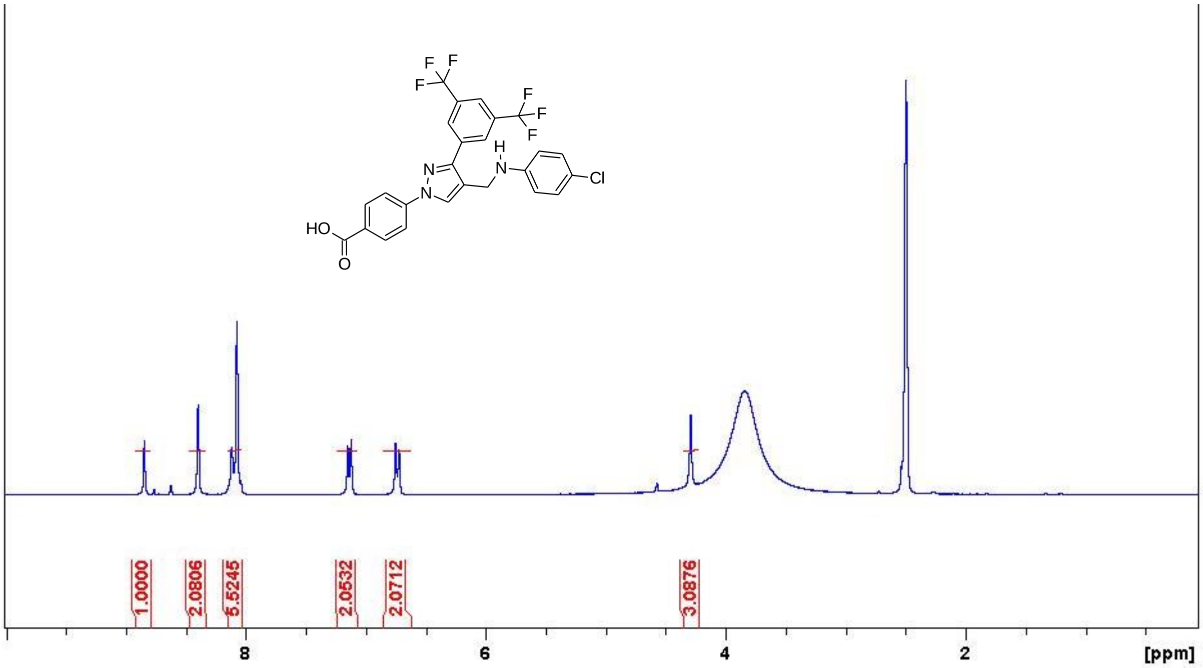

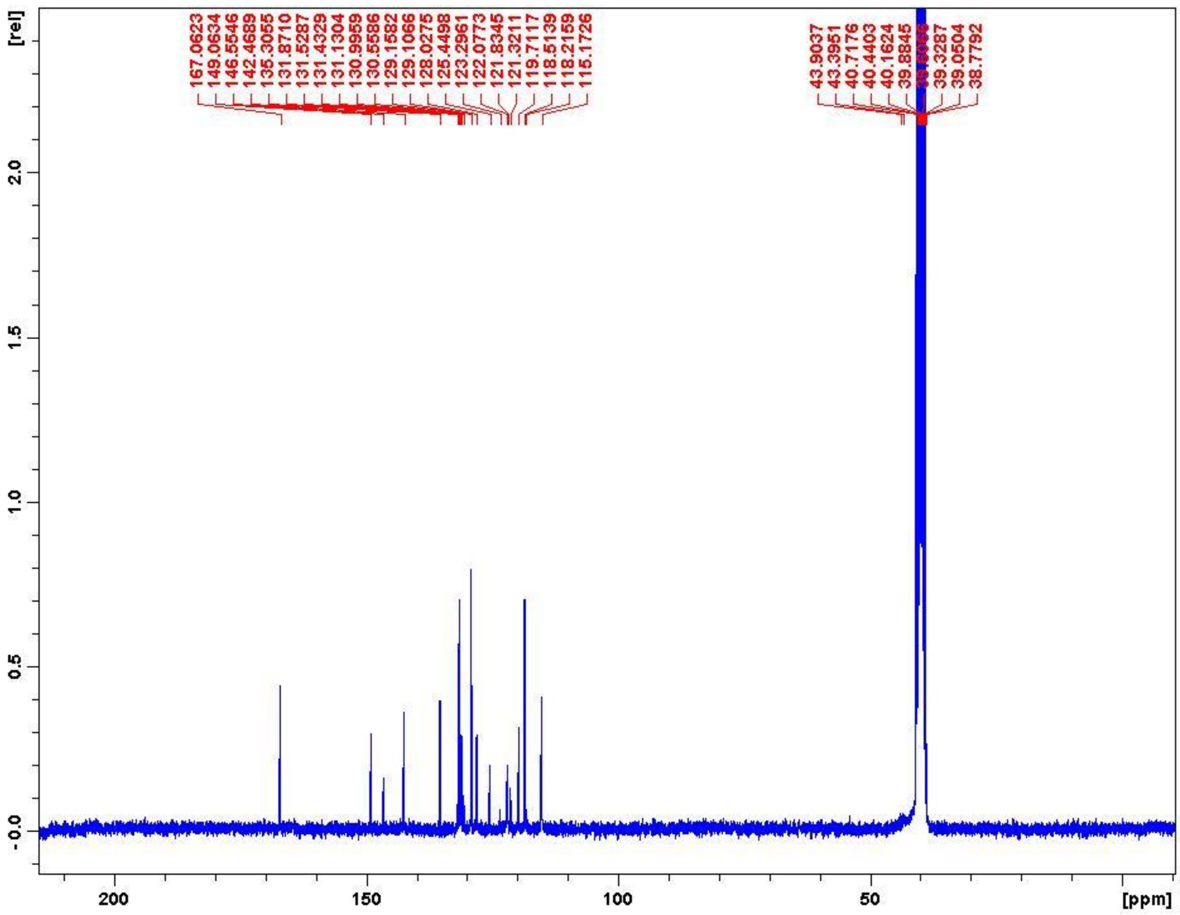

Figure S10. ^{1}H NMR and ^{13}C NMR spectra of compound **9**

4-[3-[3,5-Bis(trifluoromethyl) phenyl]-4-[(3-bromoanilino) methyl] pyrazol-1-yl] benzoic acid (10).

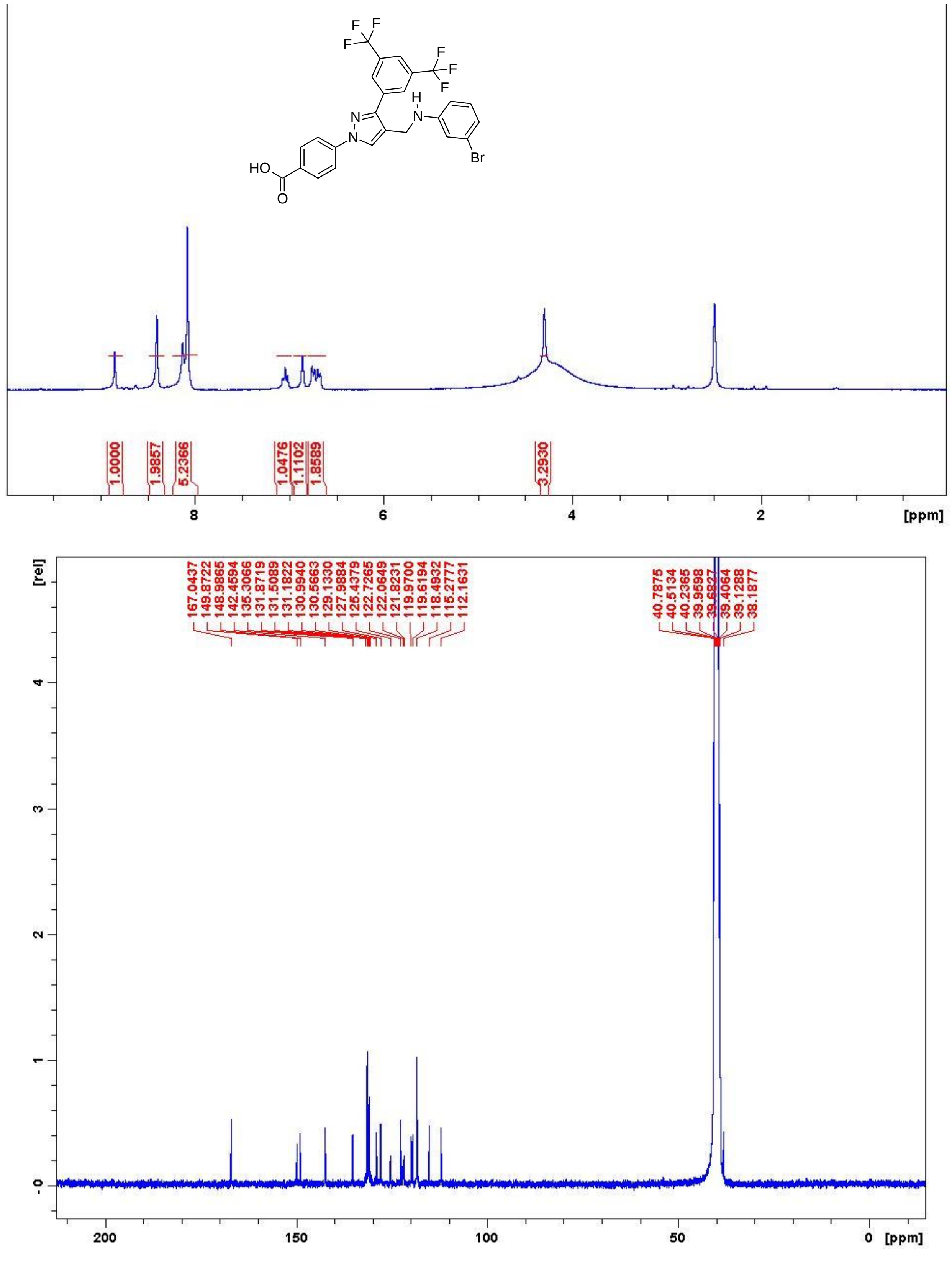

Figure S11. ^{1}H NMR and ^{13}C NMR spectra of compound **10**

4-[3-[3,5-Bis(trifluoromethyl) phenyl]-4-[(4-bromoanilino) methyl] pyrazol-1-yl] benzoic acid (11).

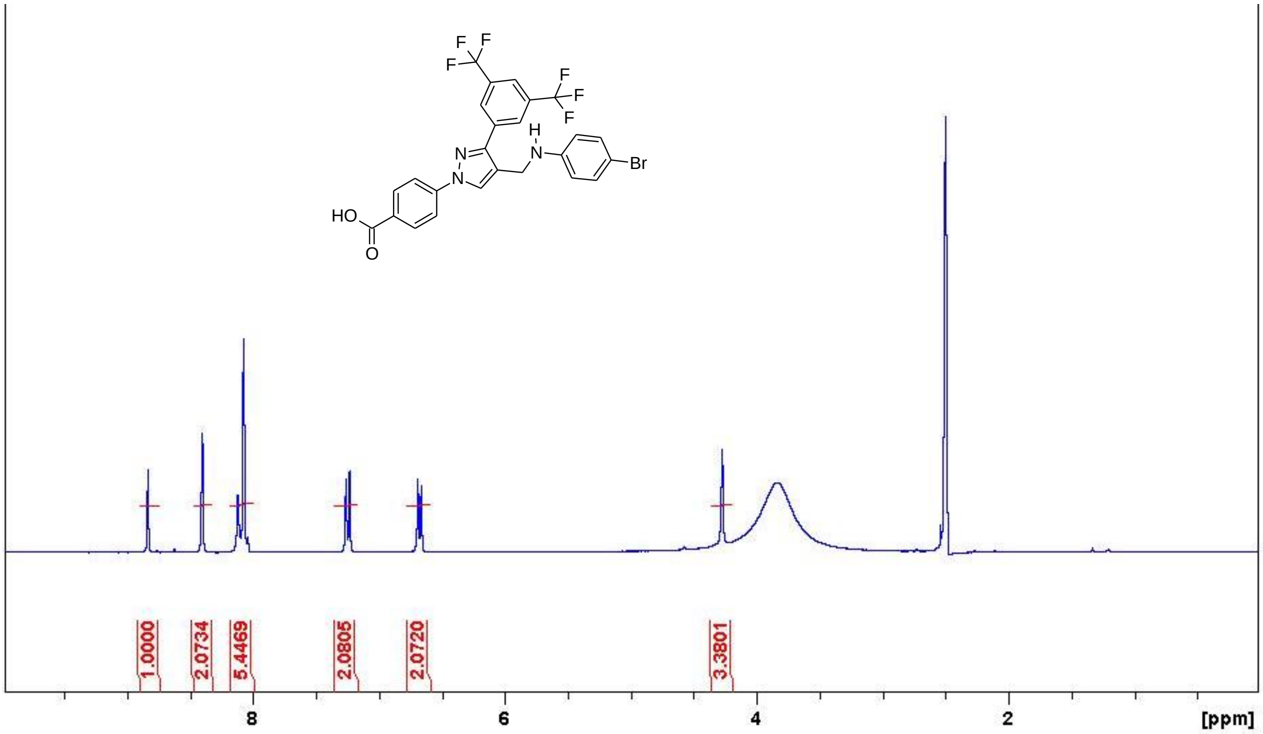

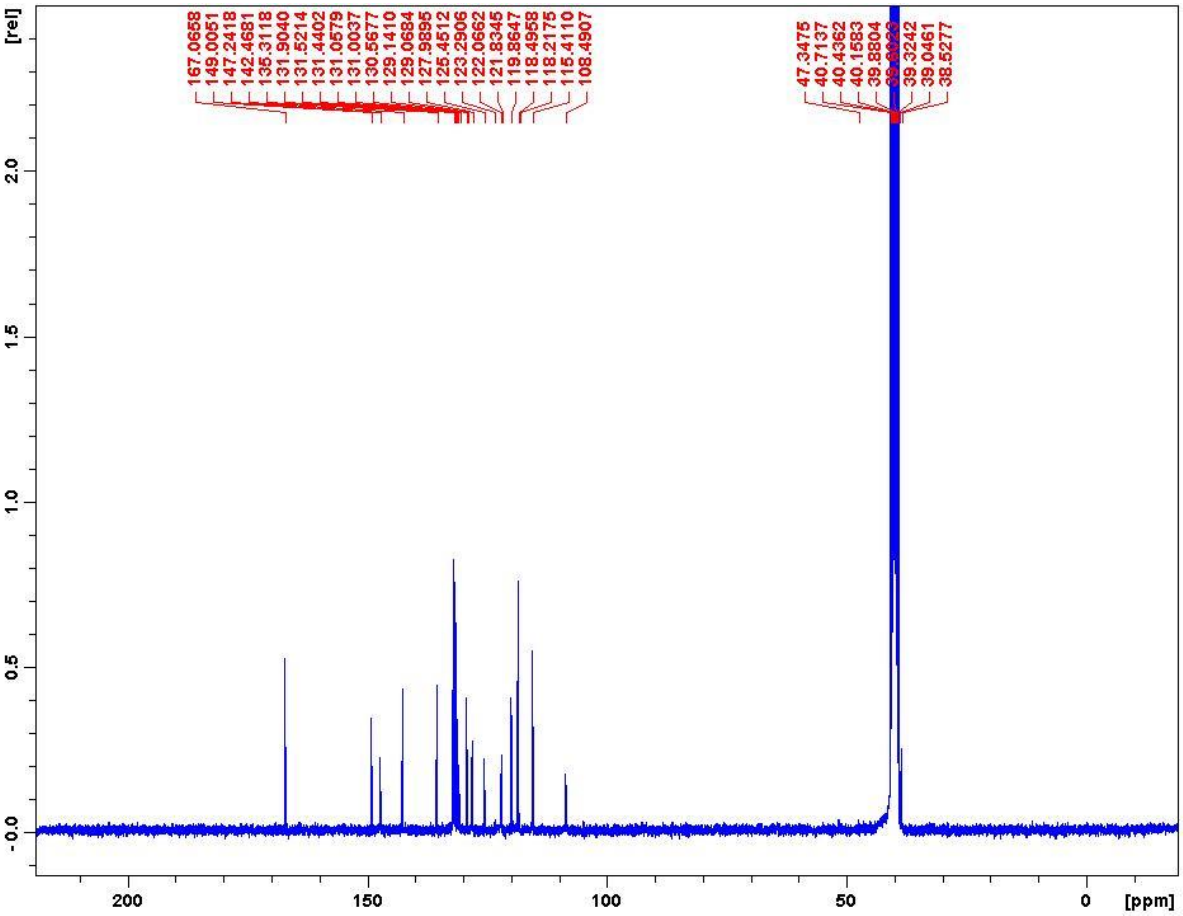

Figure S12. ^{1}H NMR and ^{13}C NMR spectra of compound **11**

3-[[3-[3,5-Bis(trifluoromethyl)phenyl]-1-(4-carboxyphenyl)pyrazol-4-yl]methylamino] benzoic acid (12).

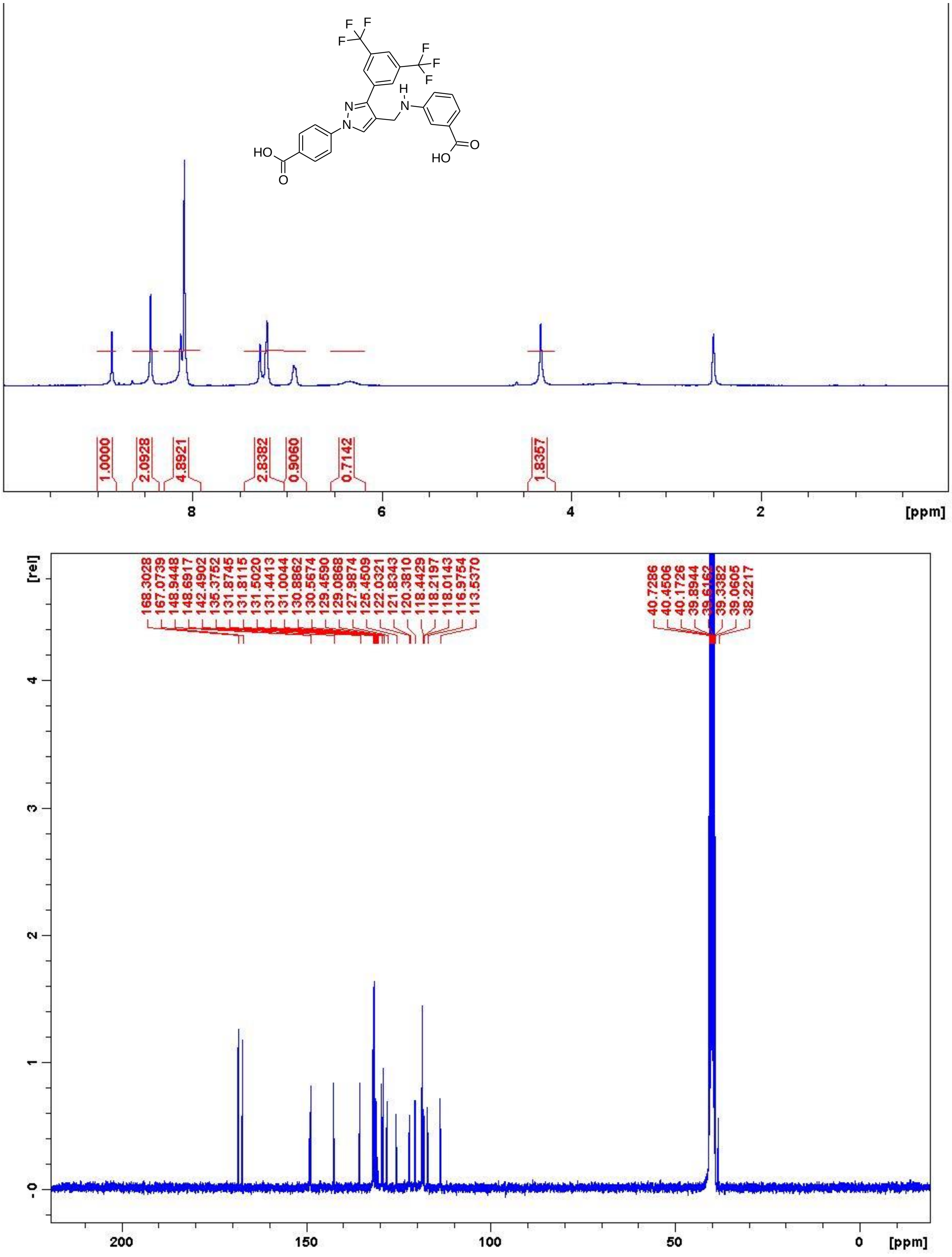

Figure S13. ^{1}H NMR and ^{13}C NMR spectra of compound **12**

4-[[3-[3,5-Bis(trifluoromethyl) phenyl]-1-(4-carboxyphenyl) pyrazol-4-yl] methylamino] benzoic acid (13).

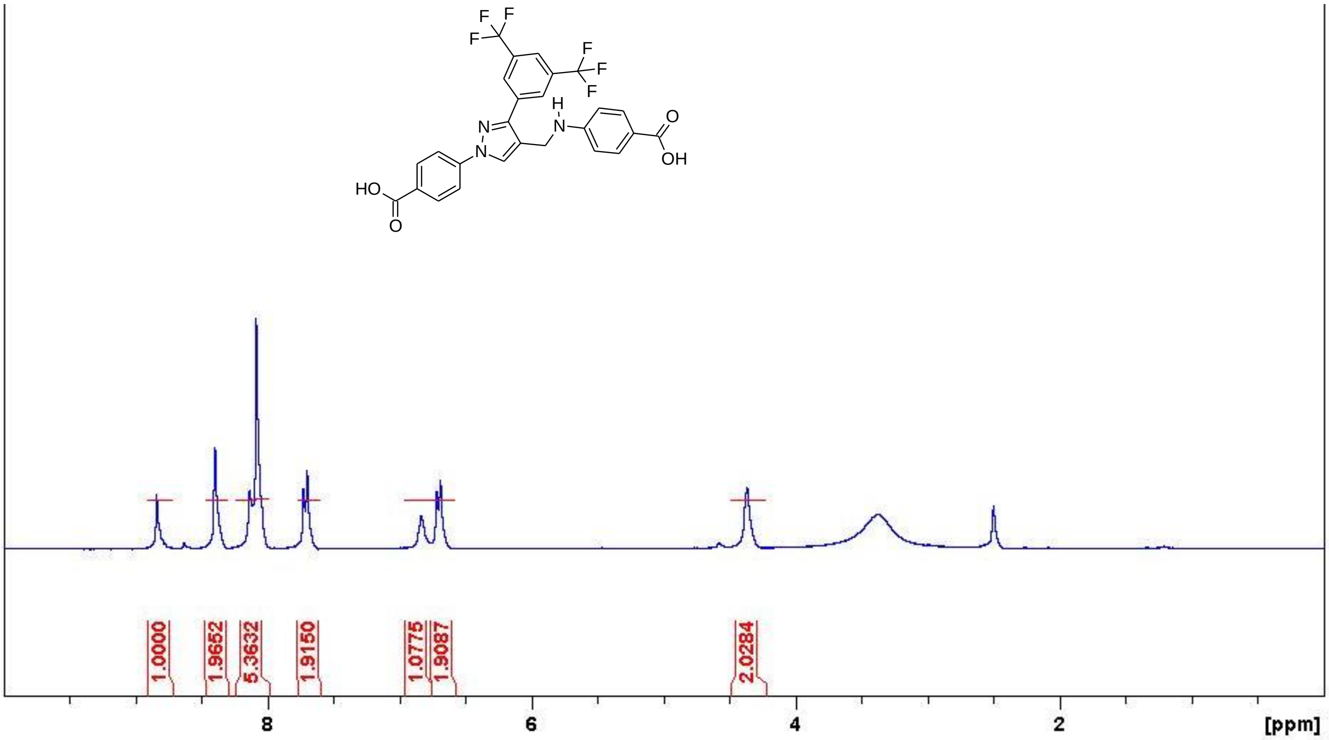

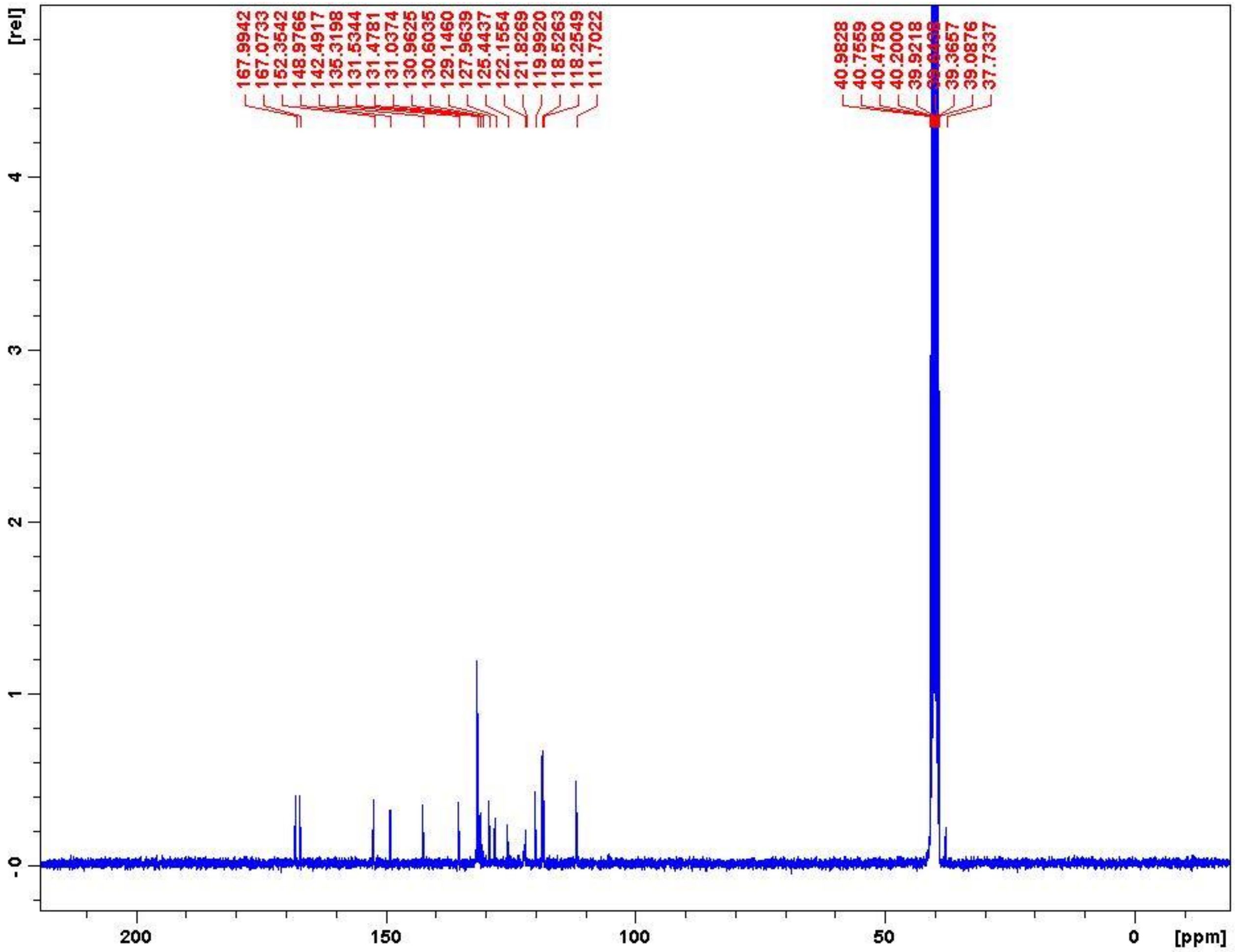

Figure S14. ^{1}H NMR and ^{13}C NMR spectra of compound **13**

4-[3-[3,5-Bis(trifluoromethyl)phenyl]-4-[(4-fluoro-3-methyl-anilino)methyl]pyrazol-1-yl] benzoic acid (14).

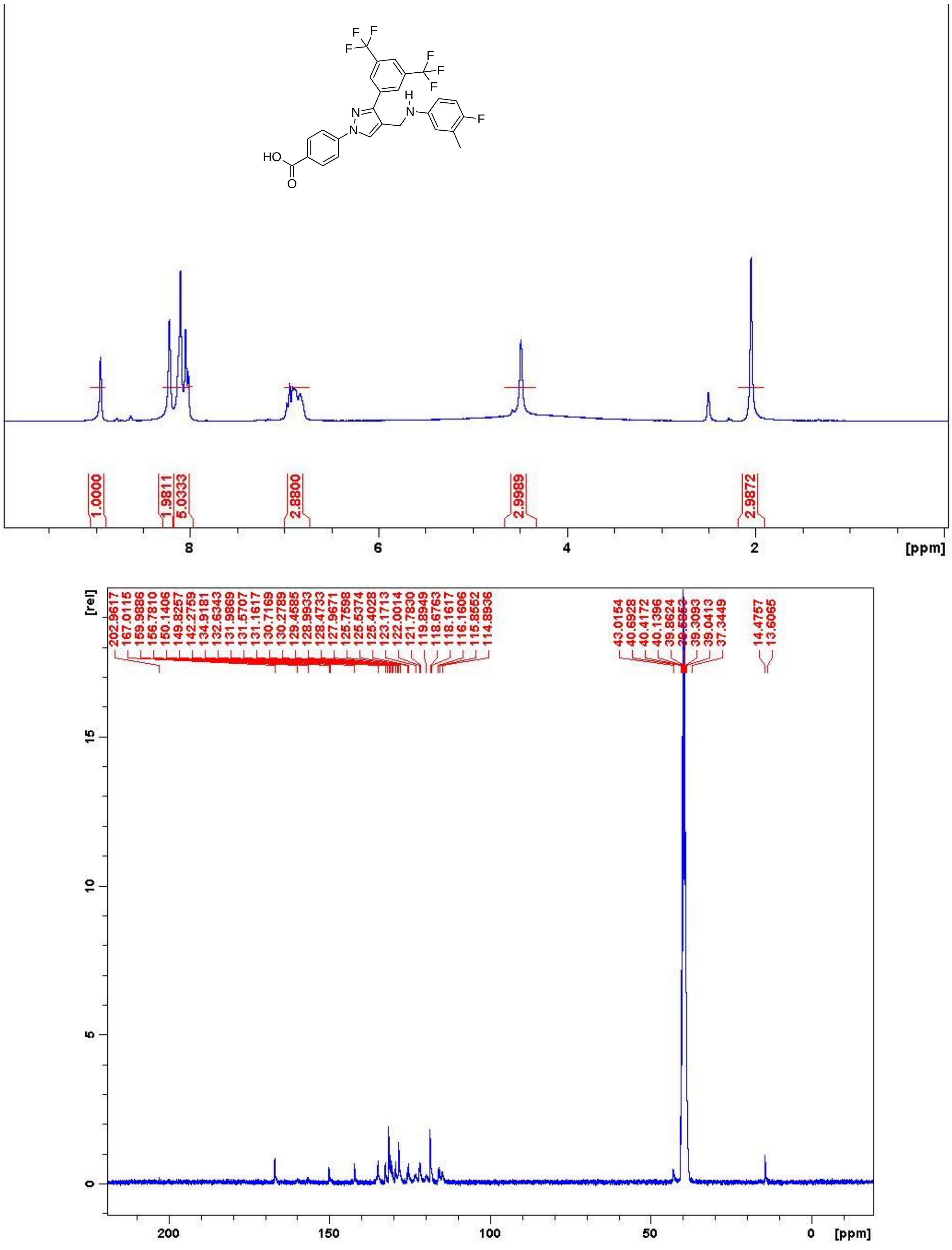

Figure S**15**. ^{1}H NMR and ^{13}C NMR spectra of compound **14**

4-[3-[3,5-Bis(trifluoromethyl)phenyl]-4-[(3-chloro-4-methyl-anilino)methyl]pyrazol-1-yl] benzoic acid (15).

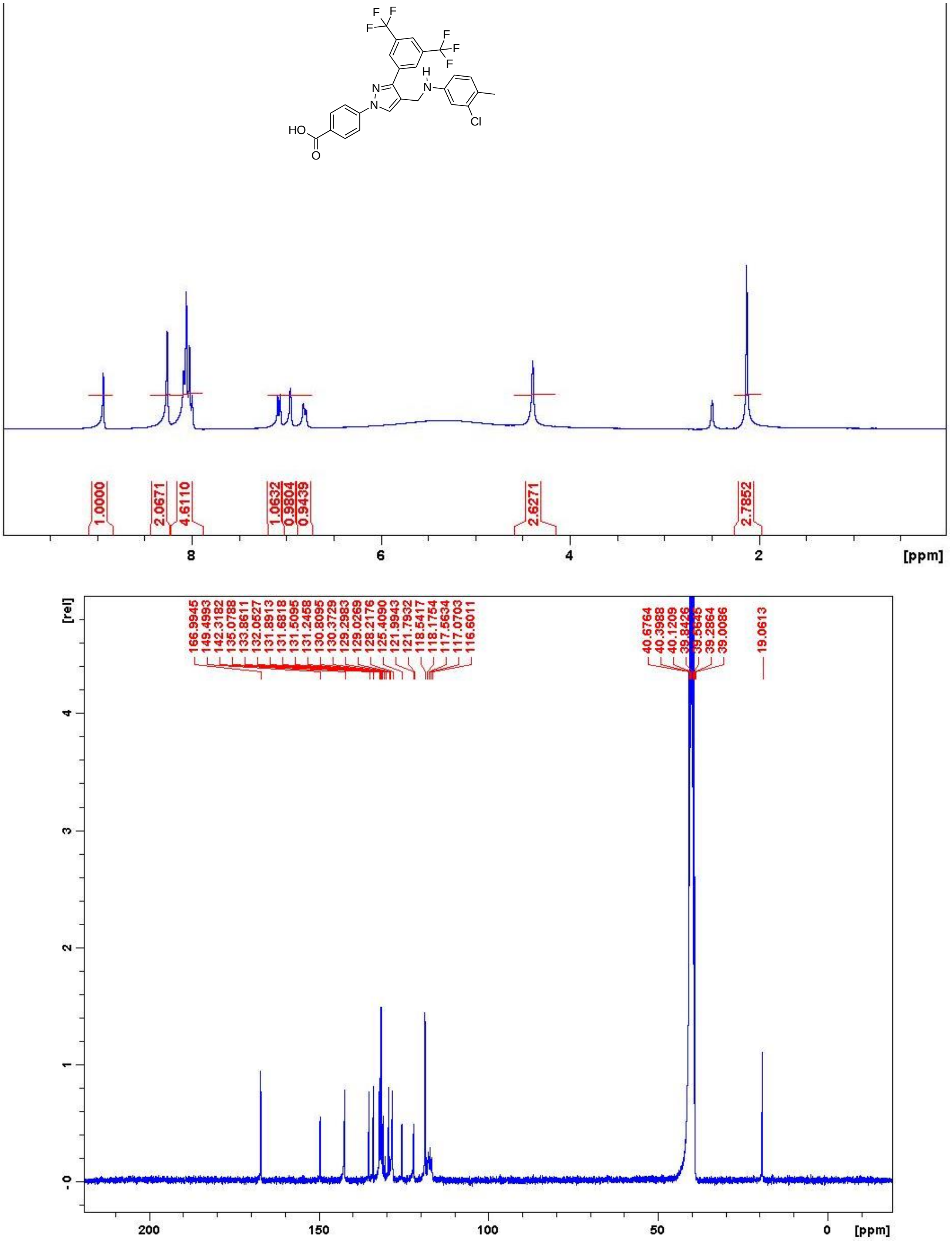

Figure S16. ^{1}H NMR and ^{13}C NMR spectra of compound **15**

4-[3-[3,5-Bis(trifluoromethyl)phenyl]-4-[(4-bromo-3-methyl-anilino)methyl]pyrazol-1-yl]benzoic acid (16).

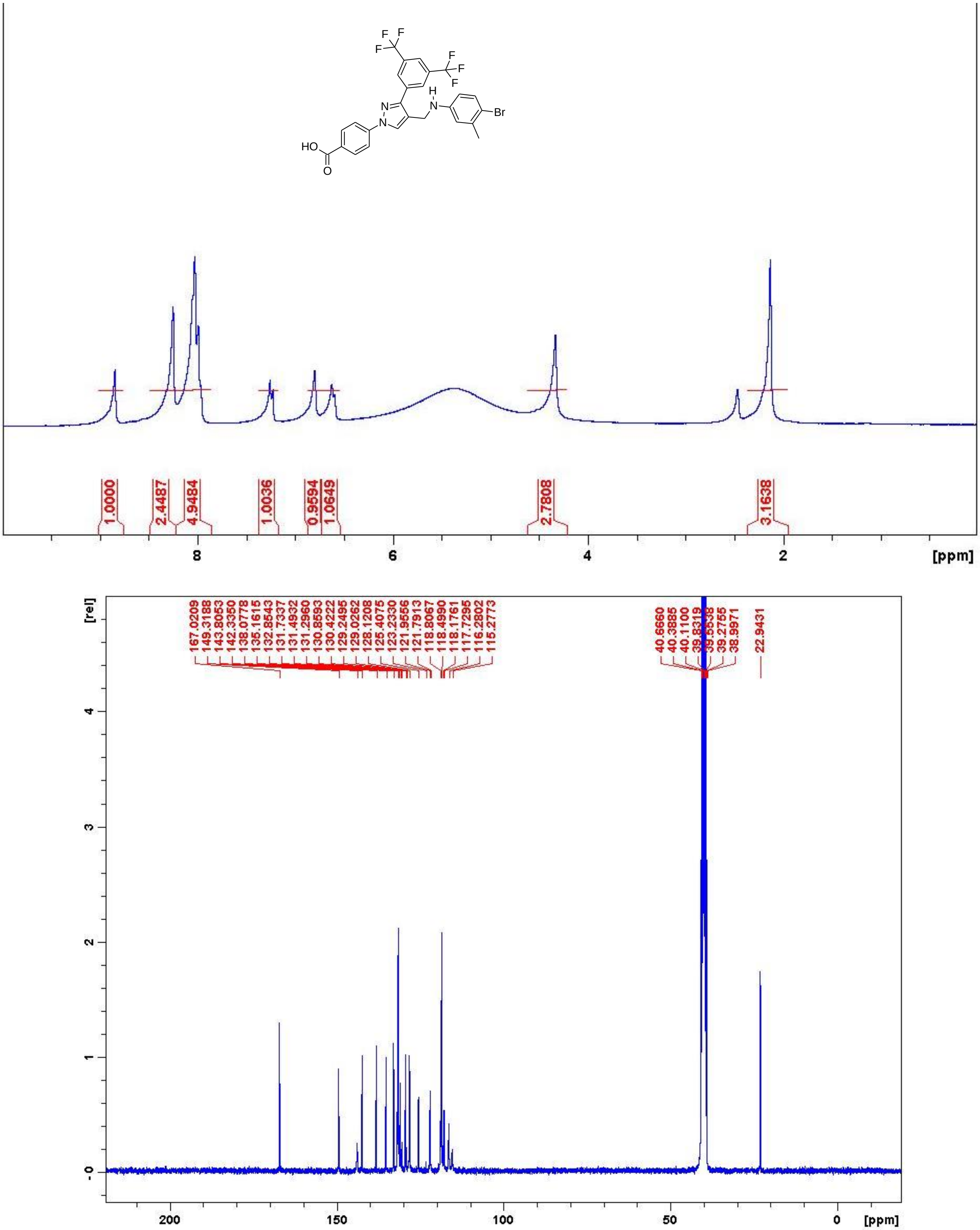

Figure S17. ^{1}H NMR and ^{13}C NMR spectra of compound **16**

4-[3-[3,5-Bis(trifluoromethyl) phenyl]-4-[(3,4-difluoroanilino) methyl] pyrazol-1-yl] benzoic acid (17).

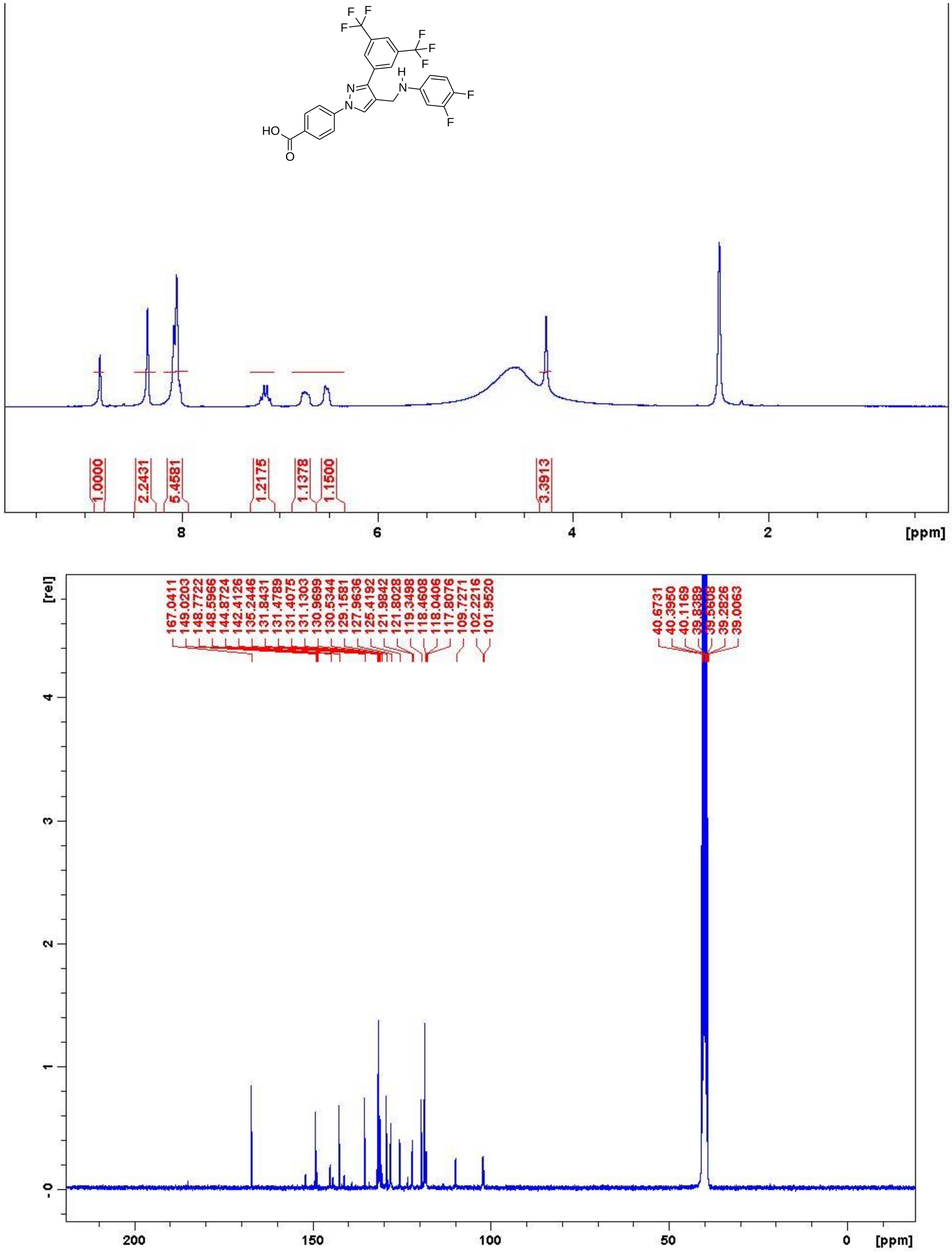

Figure S18. ^{1}H NMR and ^{13}C NMR spectra of compound **17**

4-[3-[3,5-Bis(trifluoromethyl)phenyl]-4-[(3-chloro-4-fluoro-anilino)methyl]pyrazol-1-yl] benzoic acid (18).

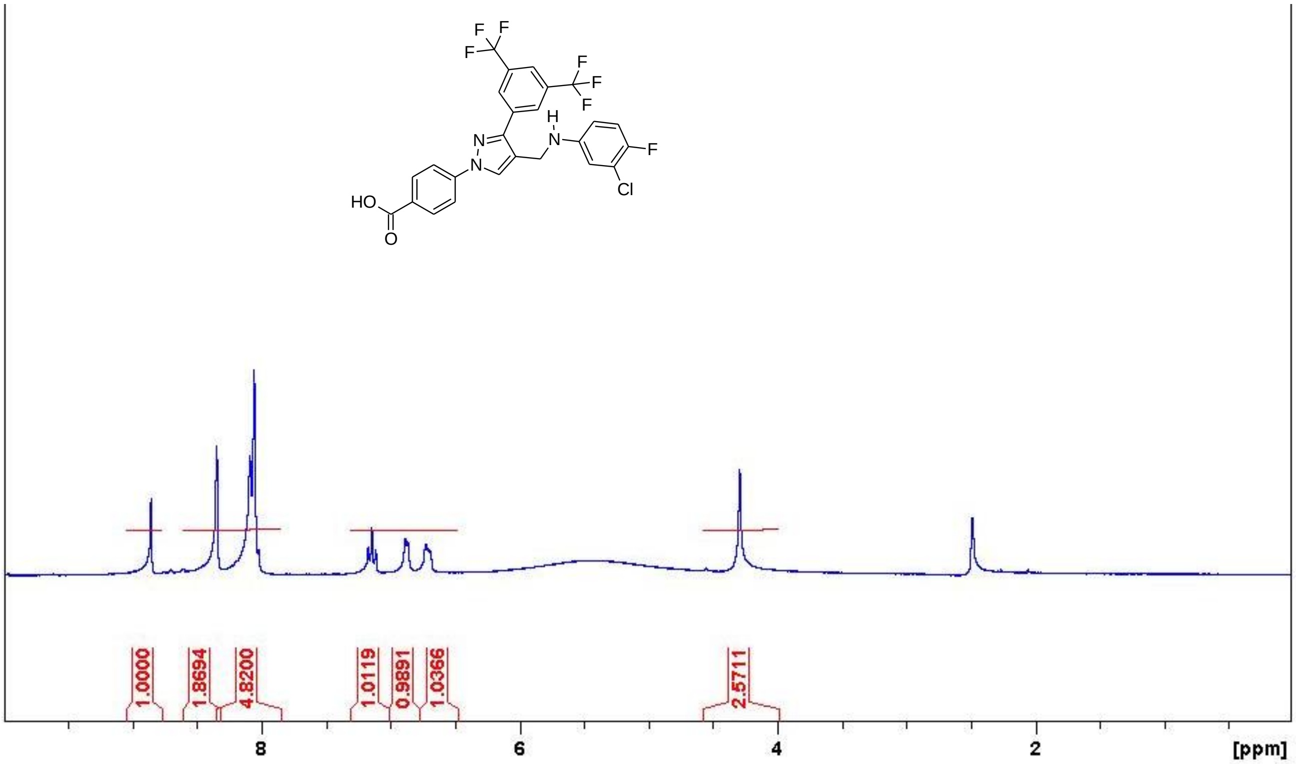

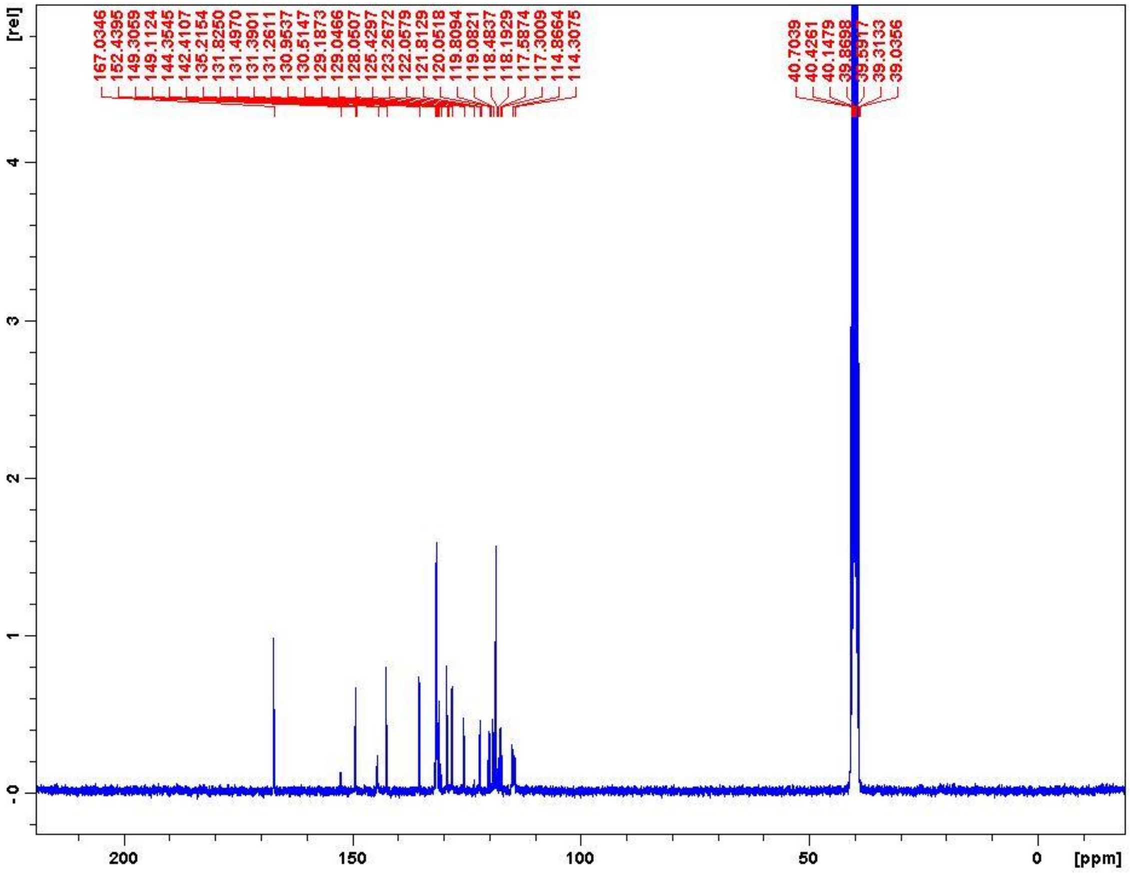

Figure S19. ^{1}H NMR and ^{13}C NMR spectra of compound **18**

4-[3-[3,5-Bis(trifluoromethyl)phenyl]-4-[(3,4-dichloroanilino)methyl]pyrazol-1-yl] benzoic acid (19).

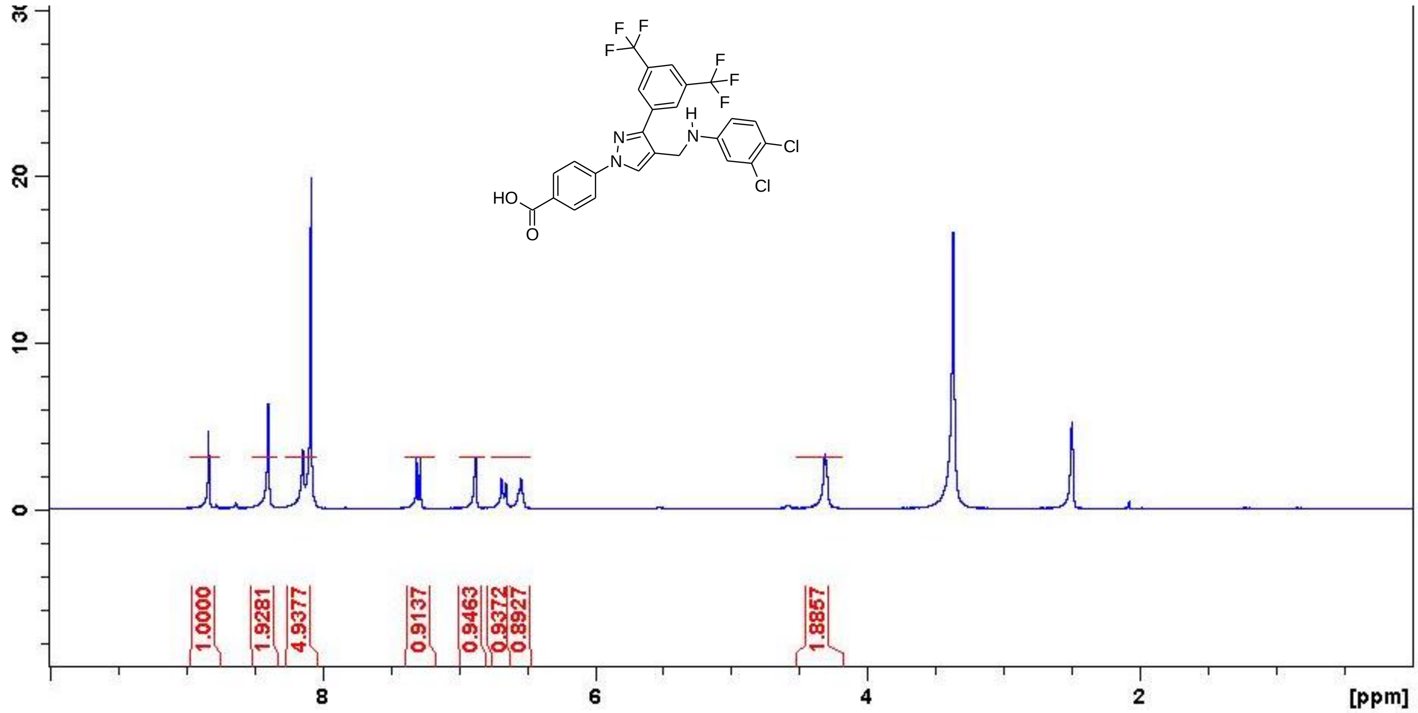

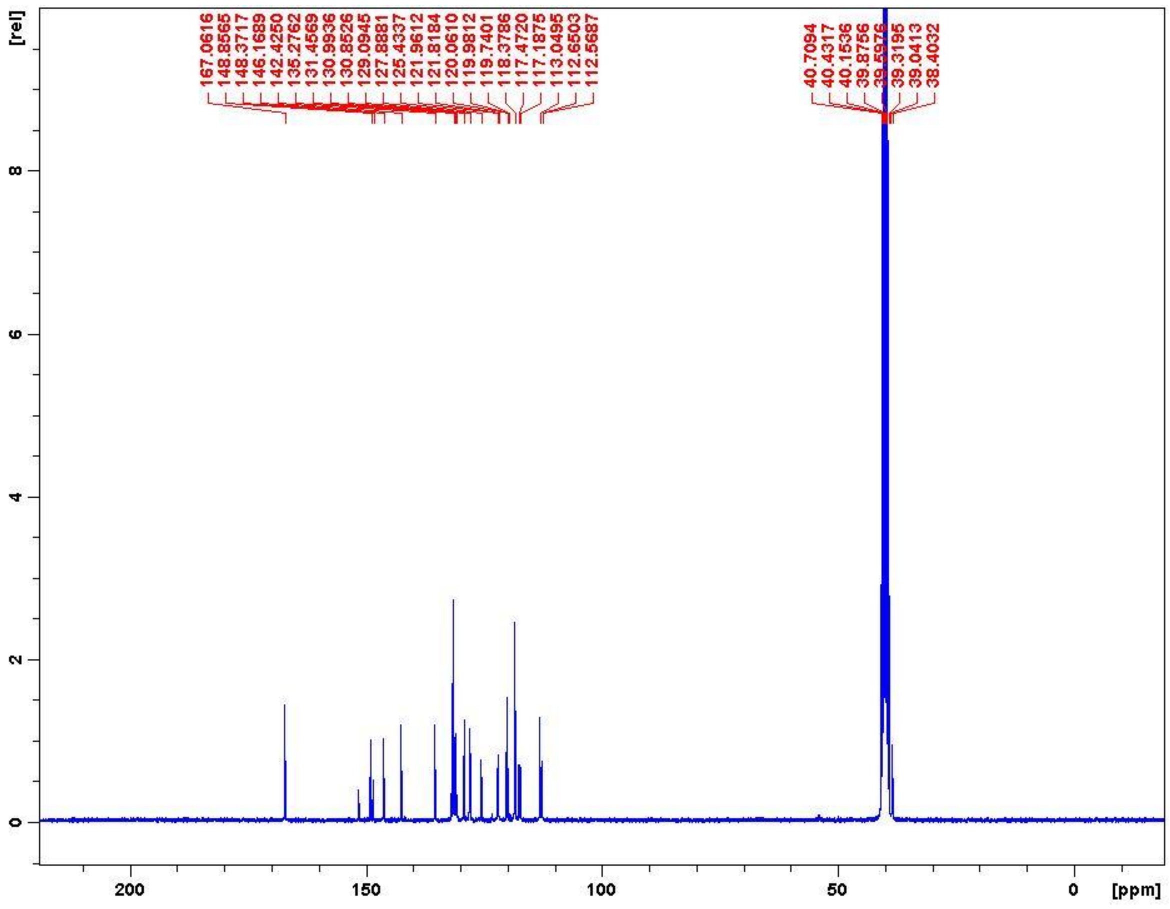

Figure S20. ^{1}H NMR and ^{13}C NMR spectra of compound **19**

4-[3-[3,5-Bis(trifluoromethyl) phenyl]-4-[(3,5-dichloroanilino) methyl] pyrazol-1-yl] benzoic acid (20).

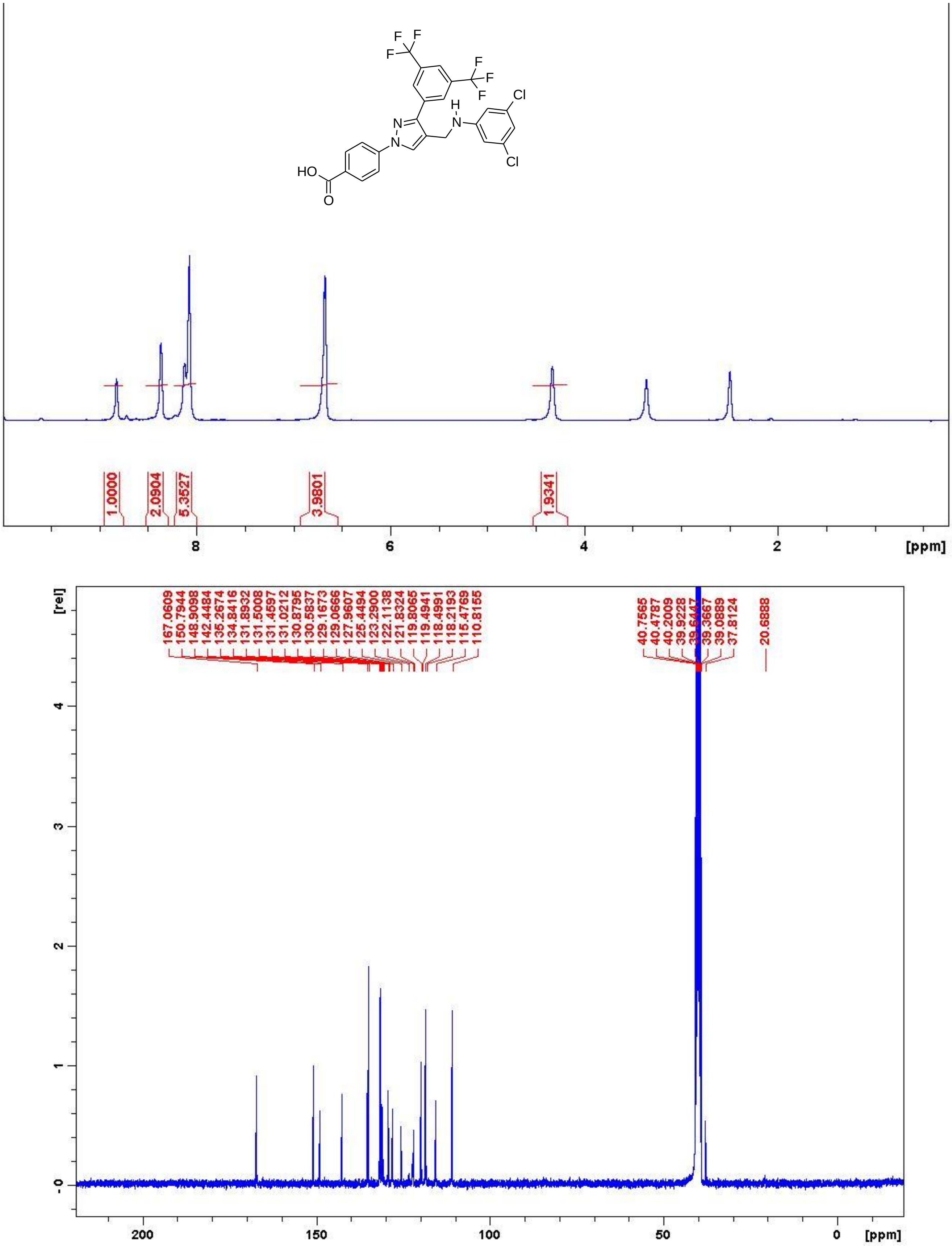

Figure S21. ^{1}H NMR and ^{13}C NMR spectra of compound **20**

4-[3-[3,5-Bis(trifluoromethyl)phenyl]-4-[(4-bromo-3-chloro-anilino)methyl]pyrazol-1-yl]benzoic acid (21).

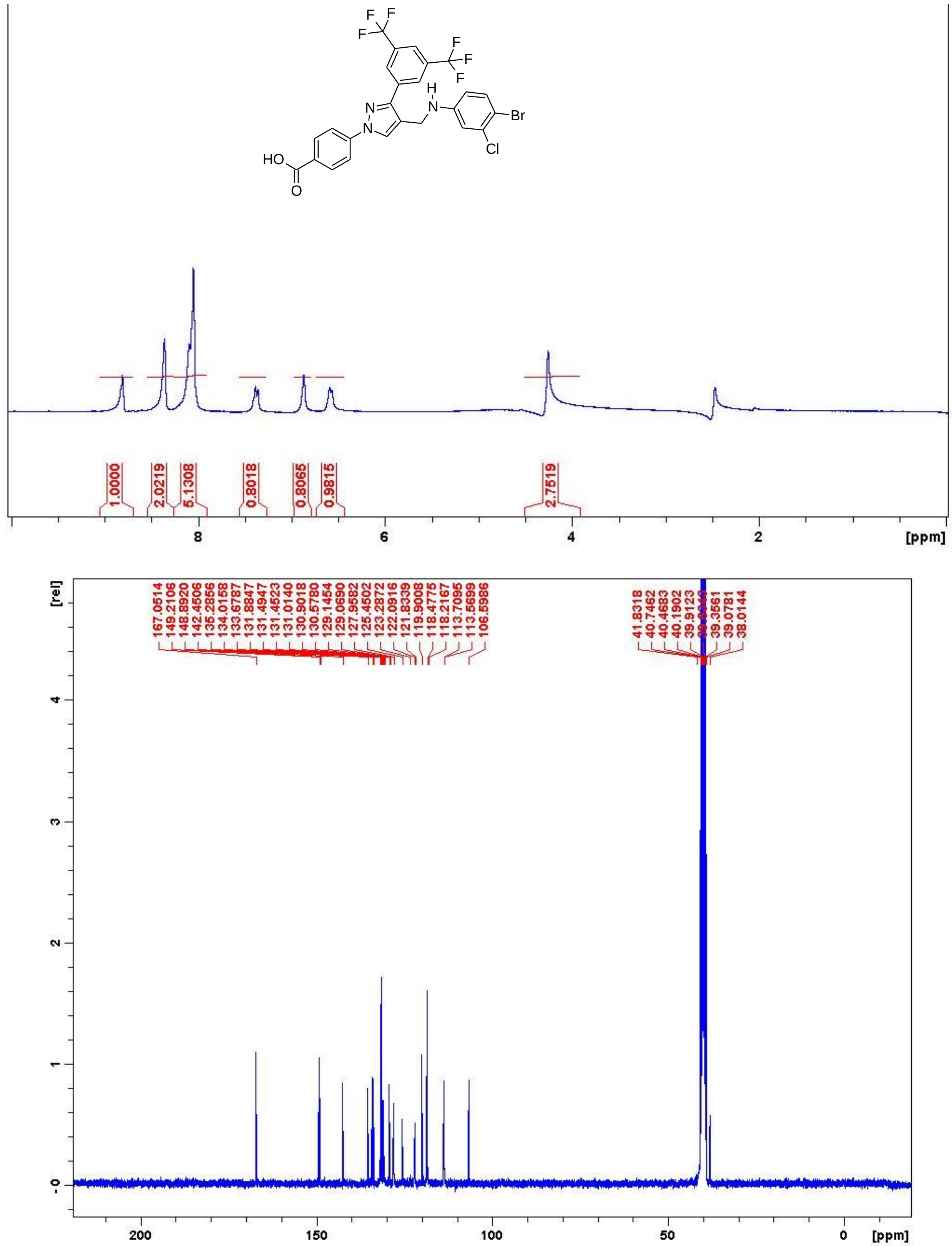

Figure S22. ^{1}H NMR and ^{13}C NMR spectra of compound **21**

4-[3-[3,5-Bis(trifluoromethyl)phenyl]-4-[[4-fluoro-3-(trifluoromethyl)anilino]methyl]pyrazol-1-yl] benzoic acid (22).

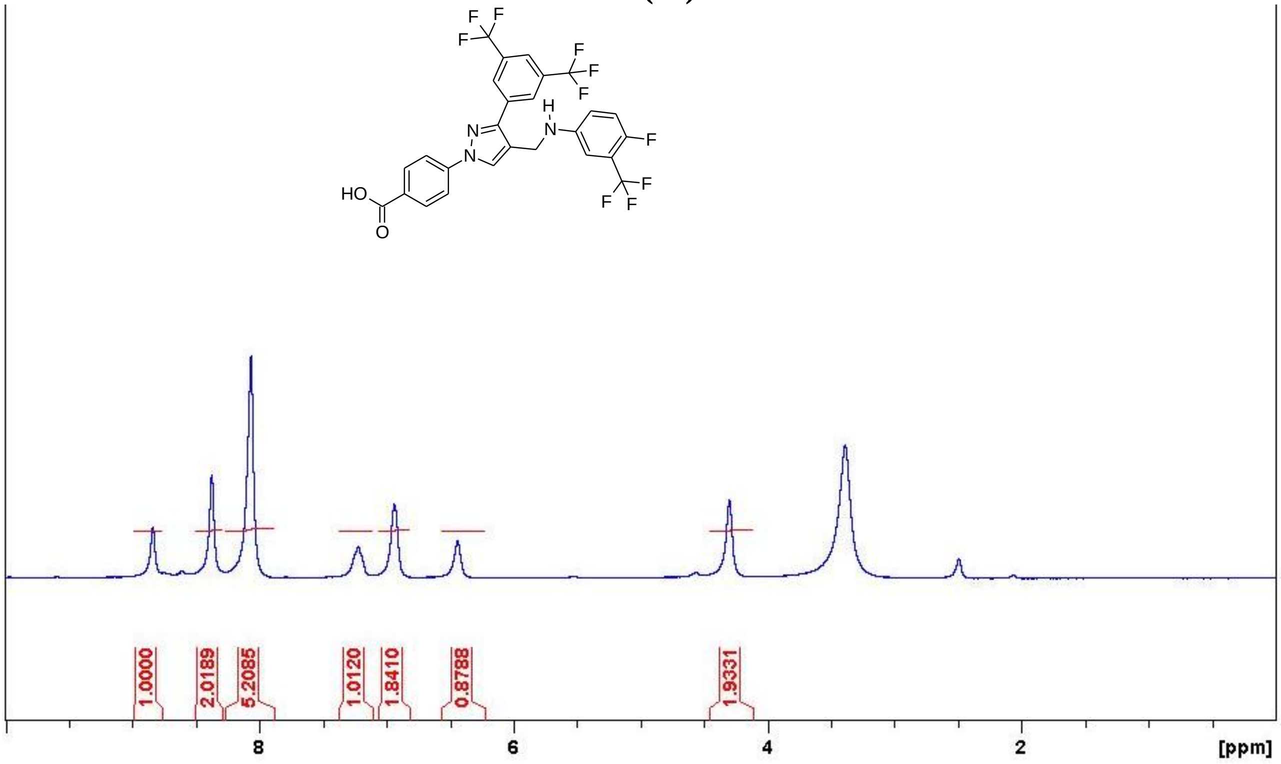

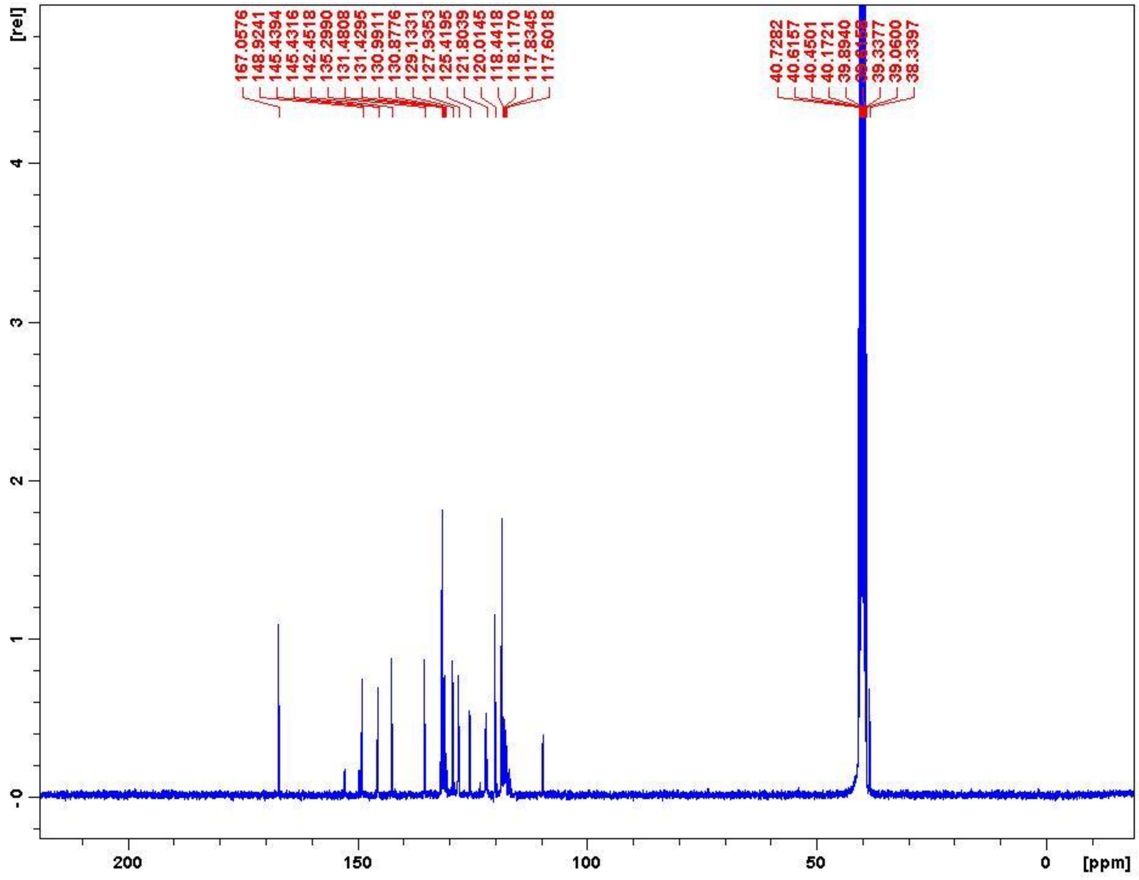

Figure S23. ^{1}H NMR and ^{13}C NMR spectra of compound **22**

4-[3-[3,5-Bis(trifluoromethyl)phenyl]-4-[[4-bromo-3-(trifluoromethyl)anilino]methyl]pyrazol-1-yl] benzoic acid (23).

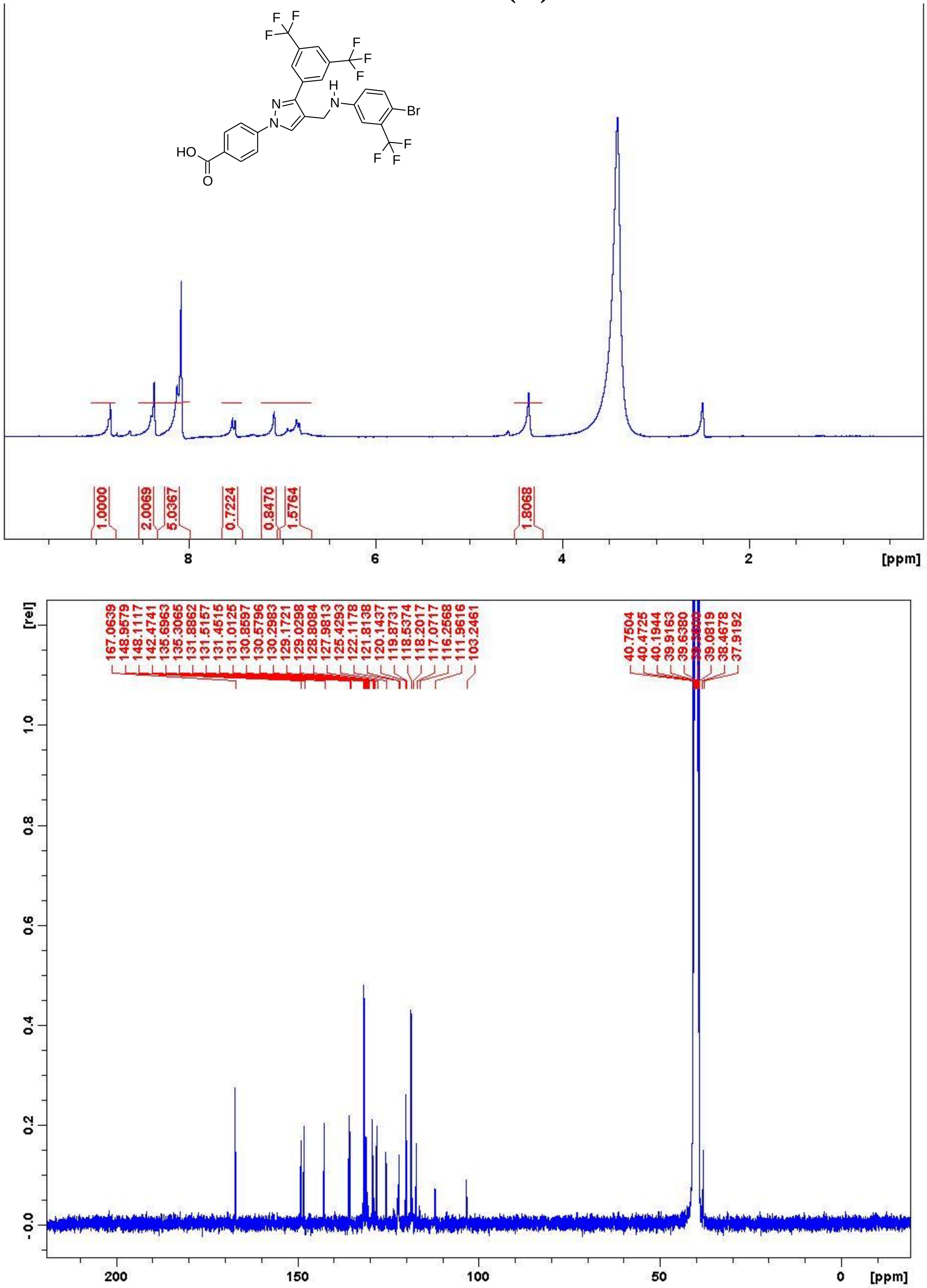

Figure S24. ^{1}H NMR and ^{13}C NMR spectra of compound **23**

4-[3-[3,5-Bis(trifluoromethyl) phenyl]-4-[(2,3,4-trifluoroanilino) methyl] pyrazol-1-yl] benzoic acid (24).

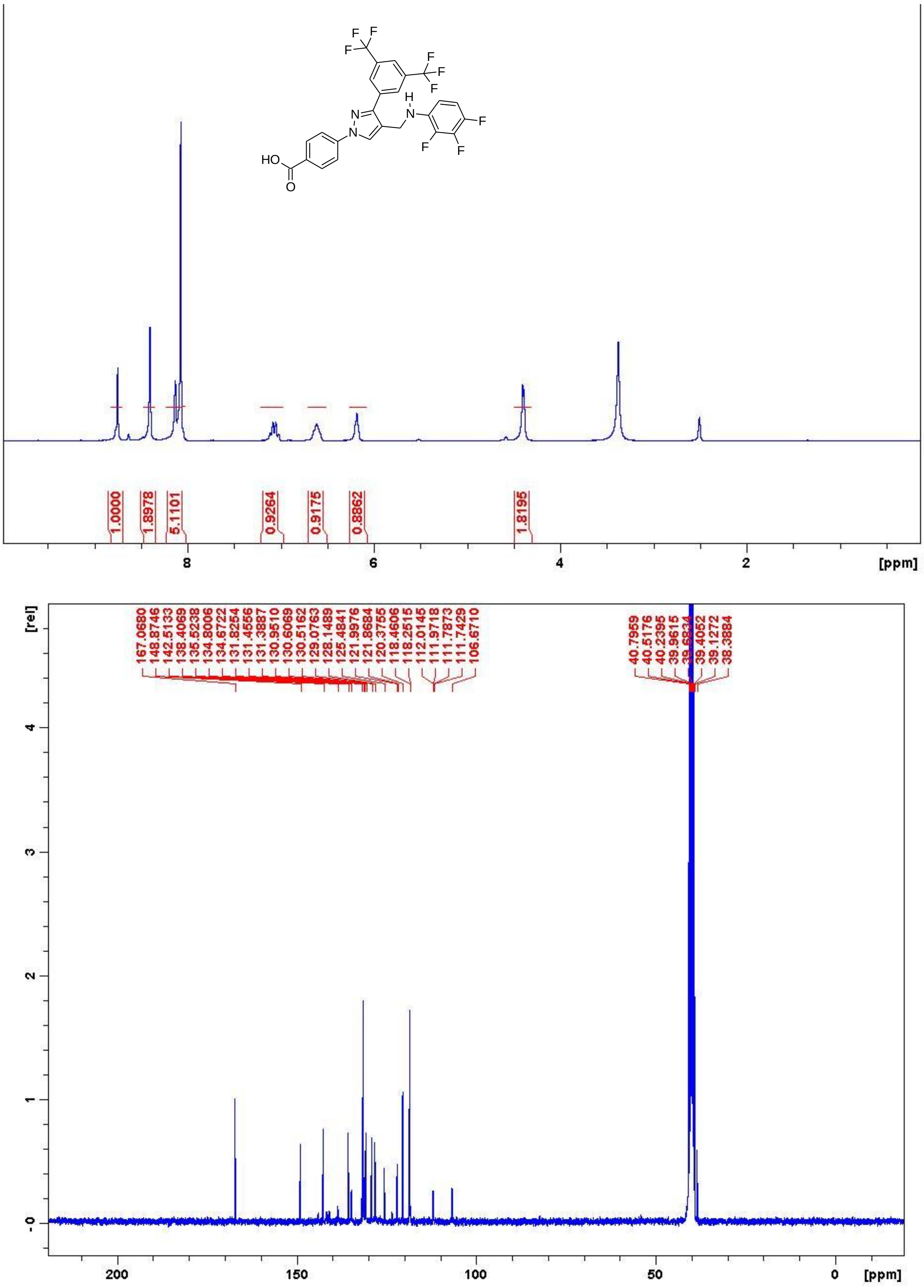

Figure S25. ^{1}H NMR and ^{13}C NMR spectra of compound **24**

4-[3-[3,5-Bis(trifluoromethyl) phenyl]-4-[(3,4,5-trifluoroanilino) methyl]pyrazol-1-yl]benzoic acid (25).

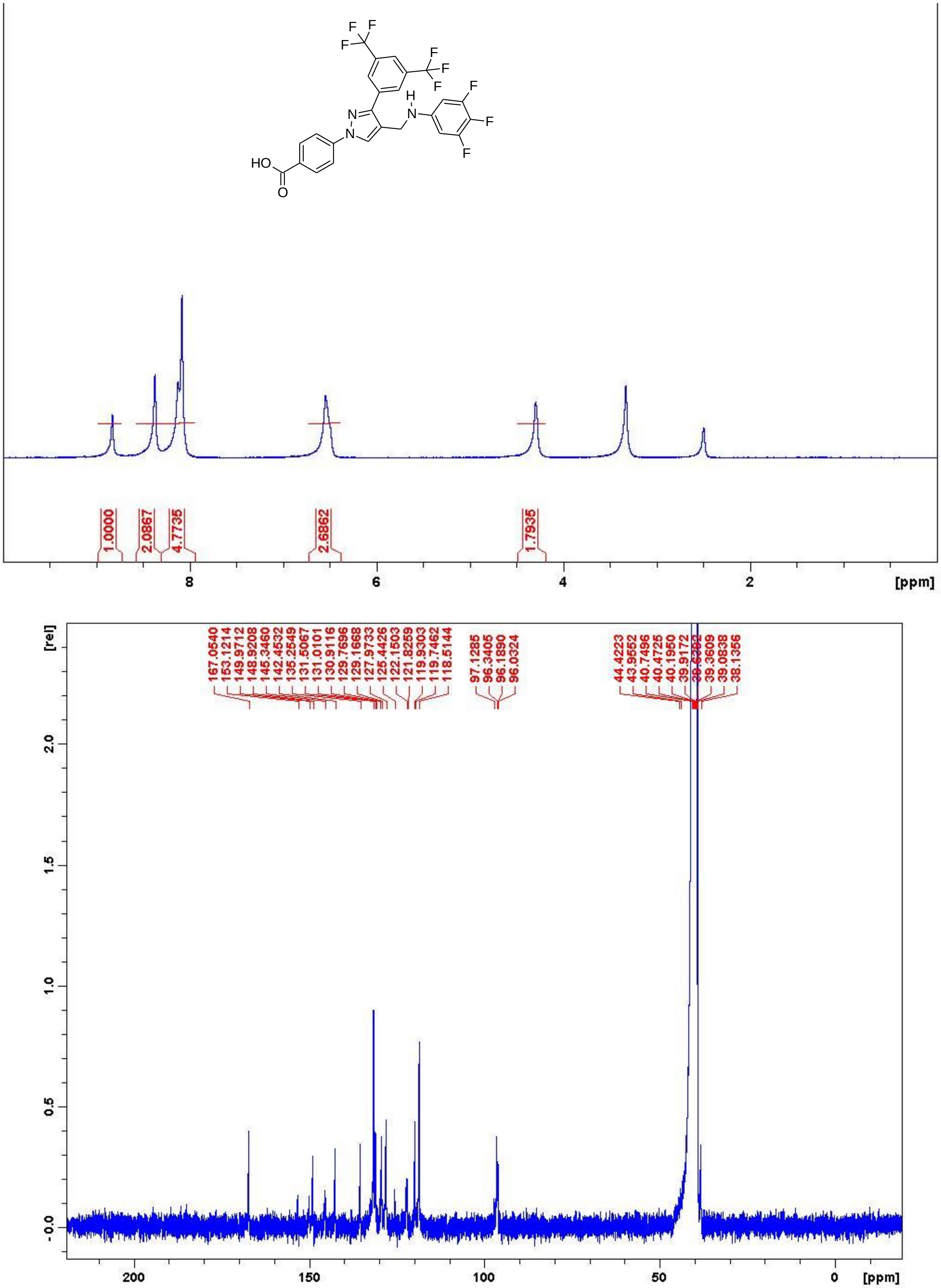

Figure S26. ^{1}H NMR and ^{13}C NMR spectra of compound **25**

4-[3-[3,5-Bis(trifluoromethyl)phenyl]-4-[(5-chloro-2,4-difluoro-anilino) methyl]pyraz-ol-1-yl] benzoic acid (26).

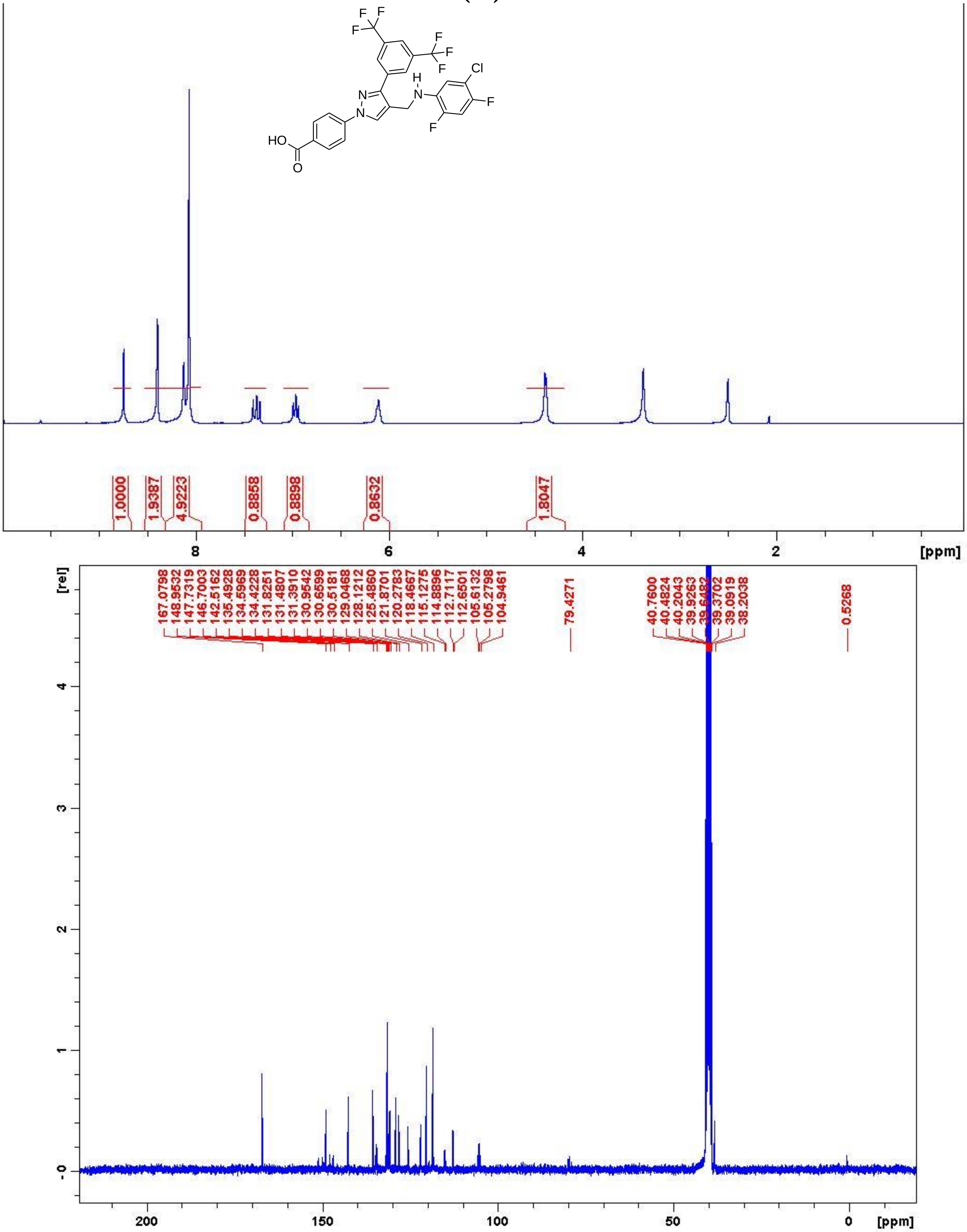

Figure S27. ^{1}H NMR and ^{13}C NMR spectra of compound **26**

4-[3-[3,5-Bis(trifluoromethyl) phenyl]-4-[(3-chloro-2,4-difluoro-anilino) methyl] pyraz-ol-1-yl] benzoic acid (27).

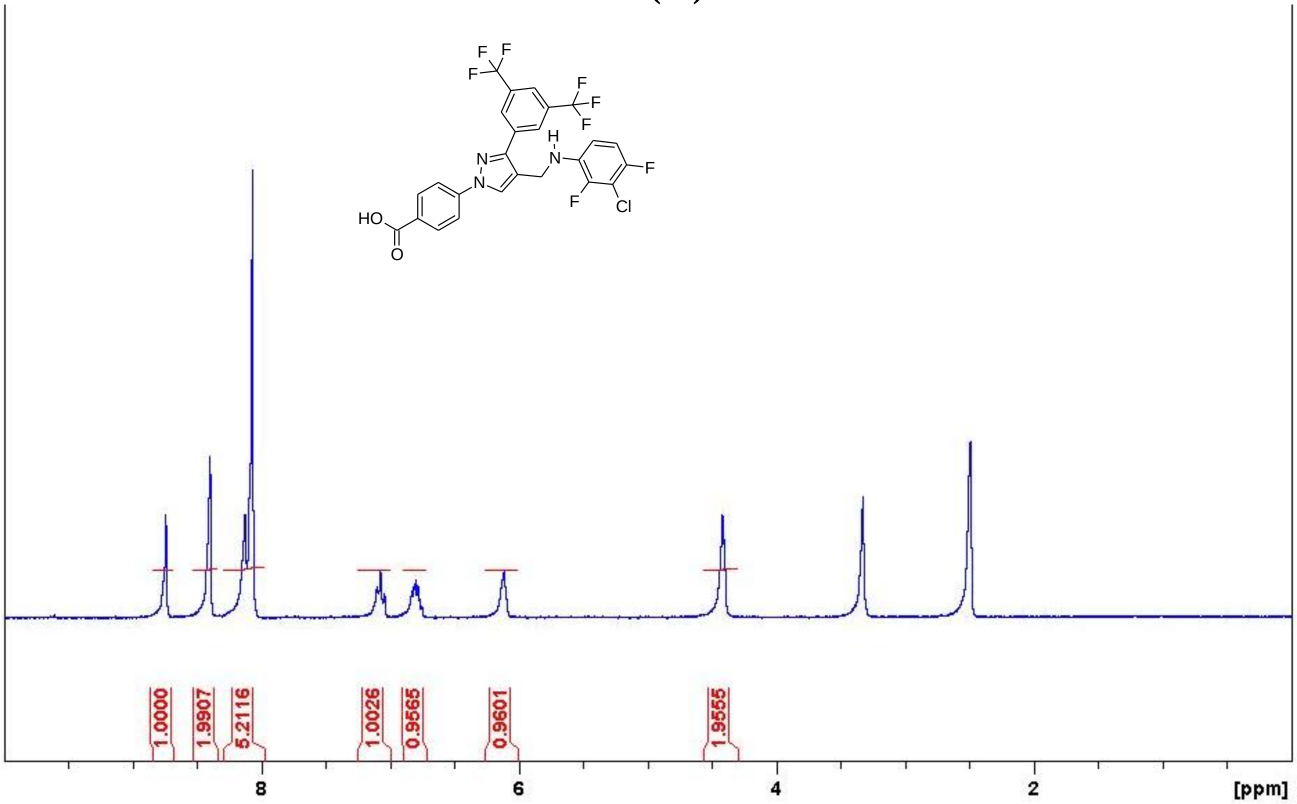

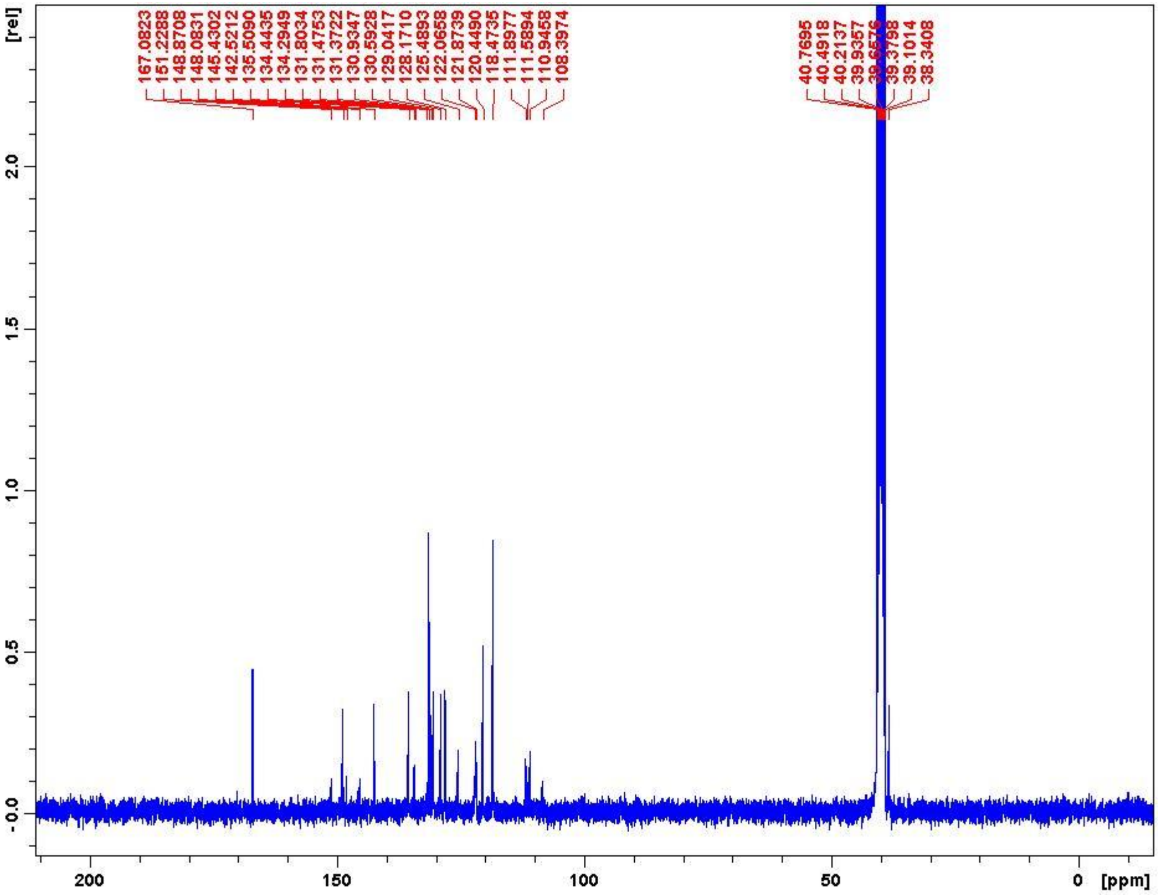

Figure S28. ^{1}H NMR and ^{13}C NMR spectra of compound **27**

4-[3-[3,5-Bis(trifluoromethyl)phenyl]-4-[(3,5-dichloro-4-fluoro-anilino)methyl]pyraz-ol-1-yl]benzoic acid (28).

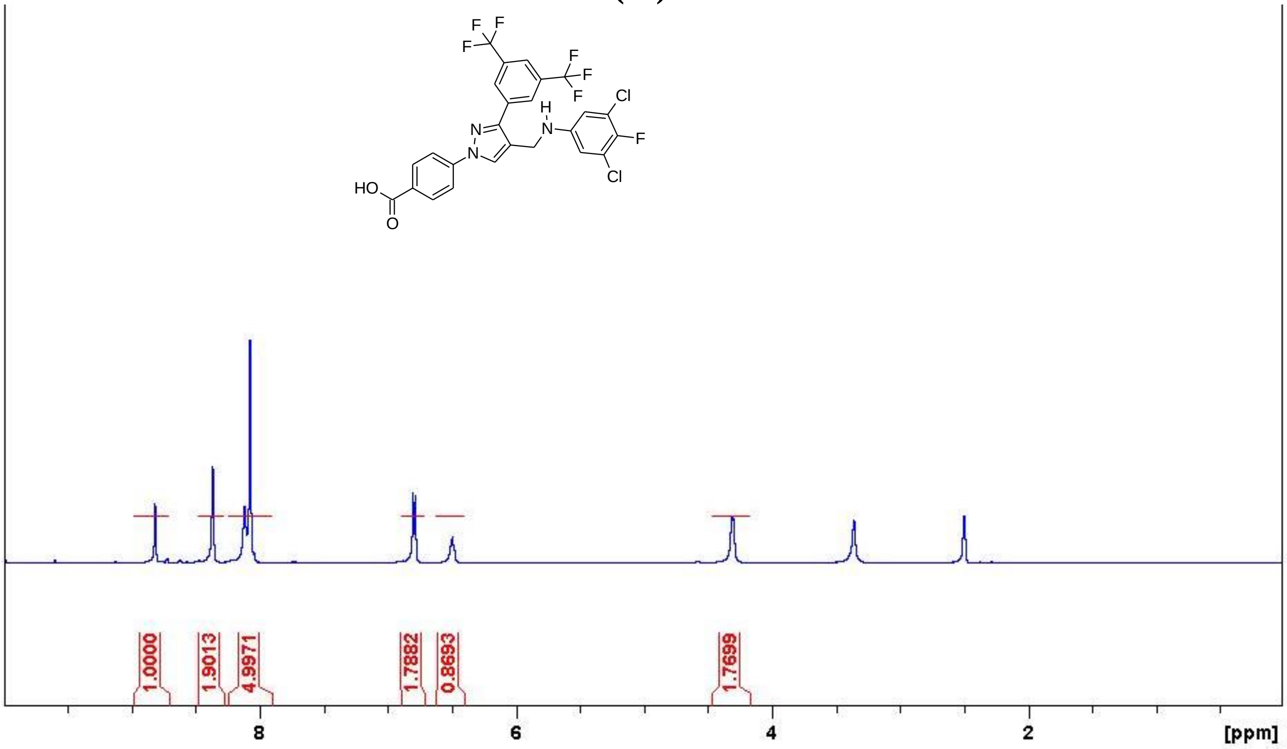

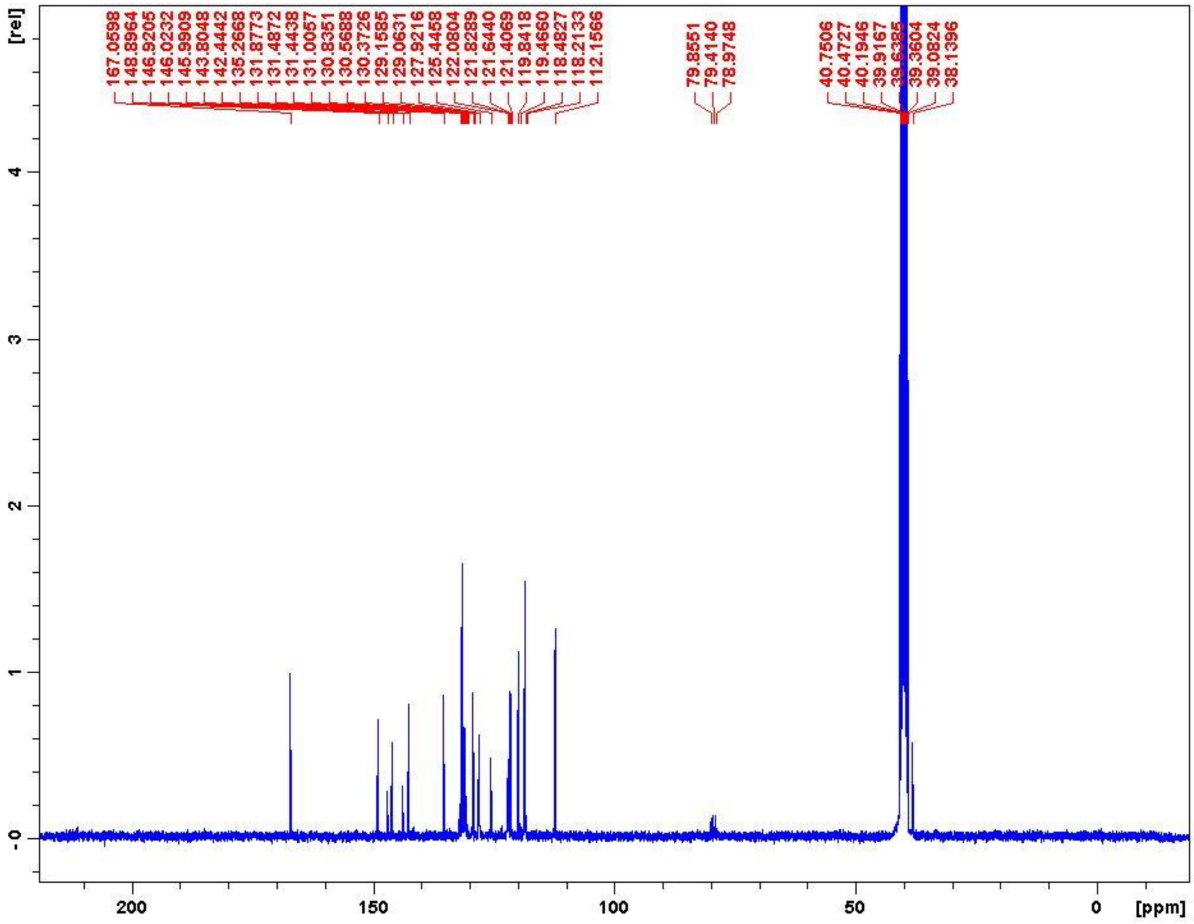

Figure S29. ^{1}H NMR and ^{13}C NMR spectra of compound **28**

4-[3-[3,5-Bis(trifluoromethyl) phenyl]-4-[(4-bromo-3-chloro-2-fluoro-anilino)methyl]pyrazol-1-yl] benzoic acid (29).

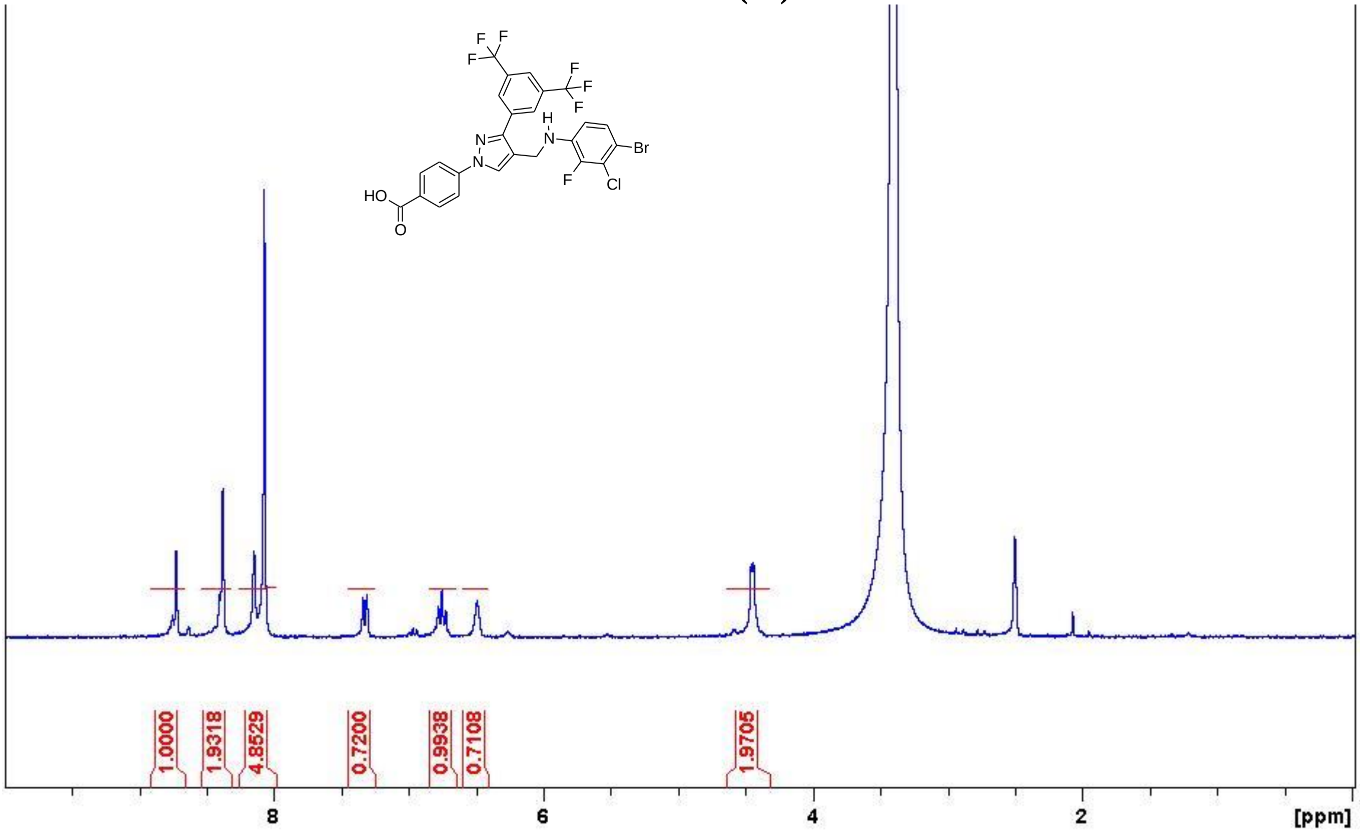

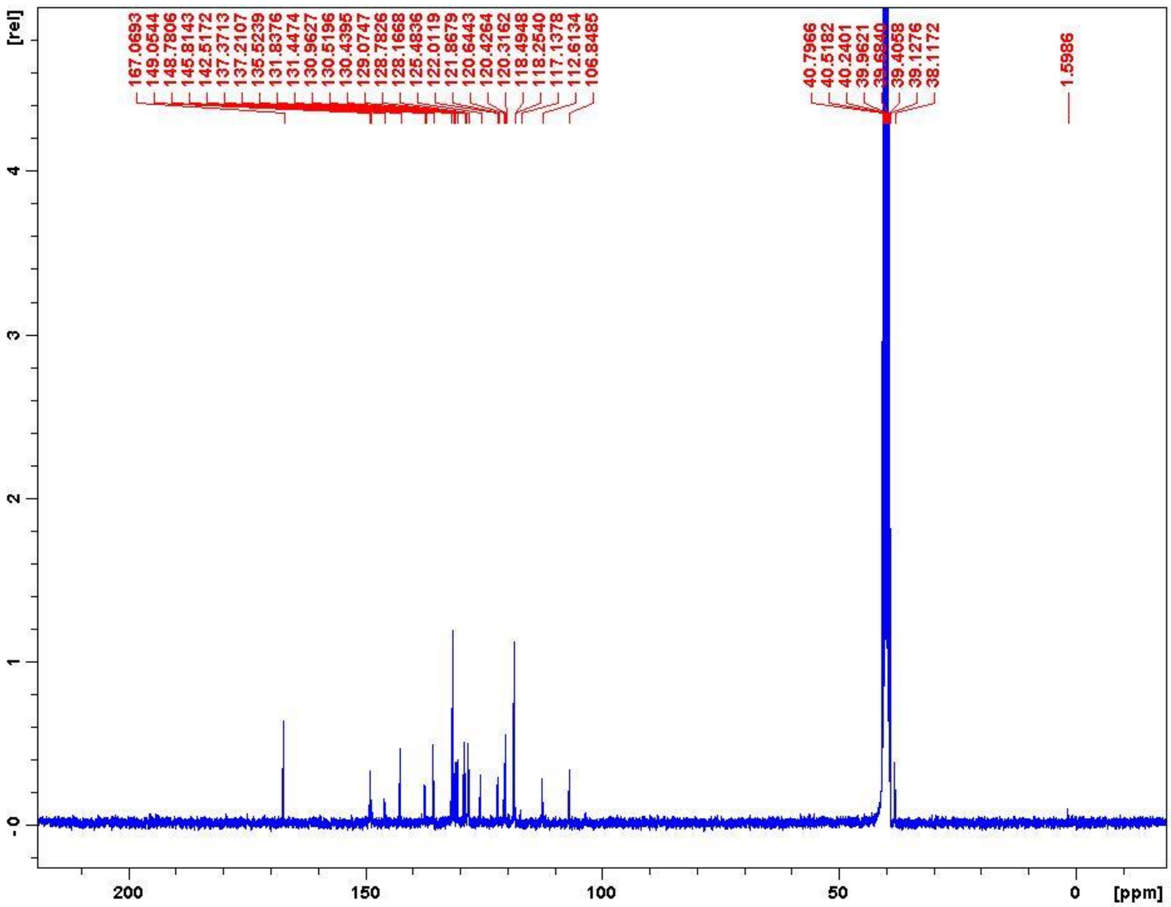

Figure S30. ^{1}H NMR and ^{13}C NMR spectra of compound **29**

4-[3-[3,5-Bis(trifluoromethyl)phenyl]-4-[(3,5-dichloro-2,4-difluoro-anilino)methyl] pyrazol-1-yl] benzoic acid (30).

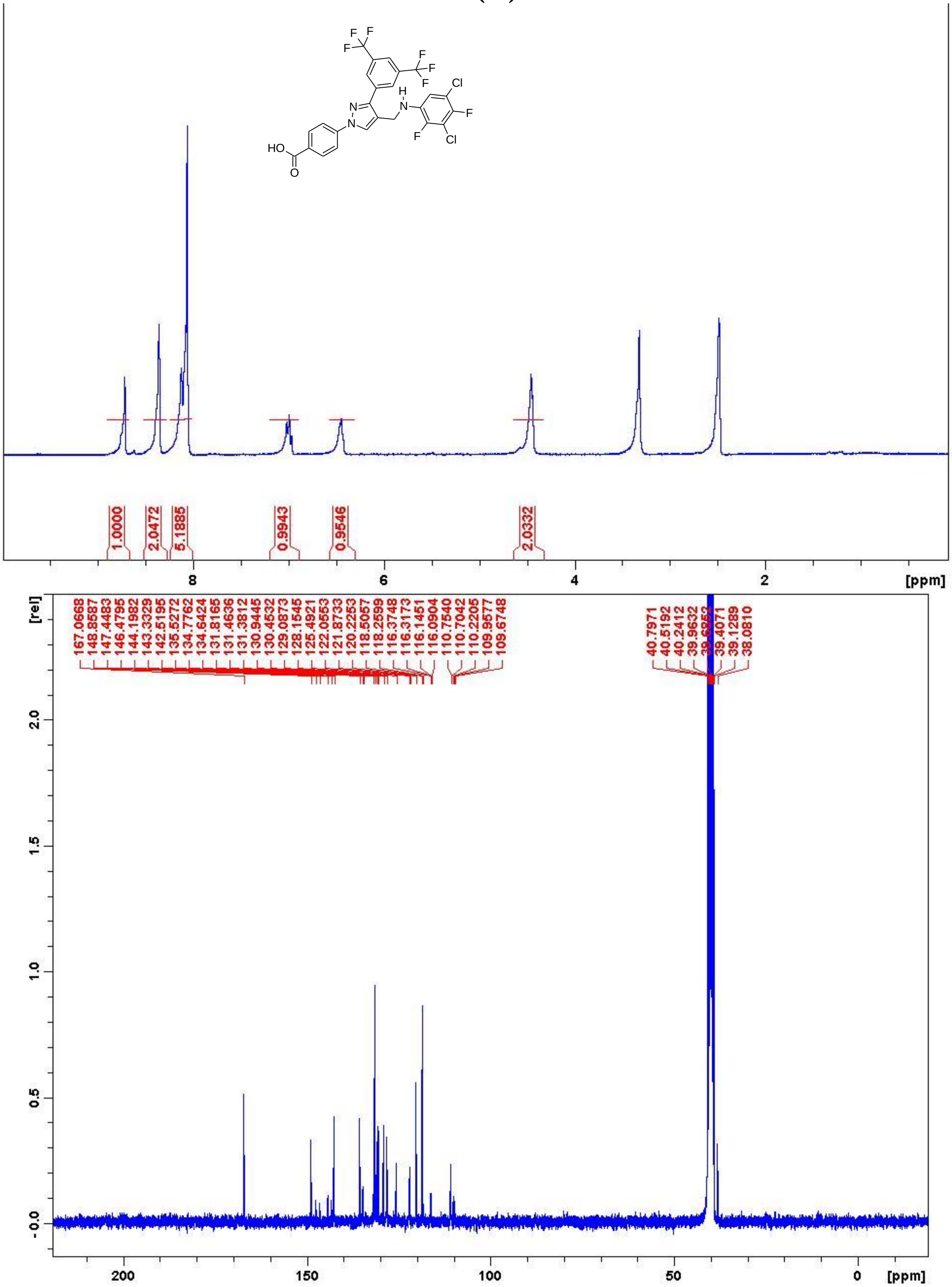

Figure S31. ^{1}H NMR and ^{13}C NMR spectra of compound **30**
